# Supplementary material for: The Two Prevalent Genotypes of an Emerging Infectious Disease, Deformed Wing Virus, Cause Equally Low Pupal Mortality and Equally High Wing Deformities in Host Honey Bees
Source: Viruses. 2019 Jan 29;11(2):114. doi: 10.3390/v11020114 (PMC6409761; doi:10.3390/v11020114)
Supplement: Supplementary file 1 [file viruses-11-00114-s001.pdf]

Supplementary Materials

Article title: The two prevalent genotypes of an emerging infectious disease, *Deformed wing virus*, cause equally low pupal mortality and equally high wing deformities in host honey bees

Authors: Anja Tehel, Quynh Vu, Diane Bigot, Andreas Gogol-Döring, Peter Koch, Christina Jenkins, Vincent Doublet, Panagiotis Theodorou, Robert Paxton

Contents:

Figure S1: NGS read coverage of the experimental inocula across the DWV genome

Figure S2: Genetic variability of the experimental inocula across the DWV genome

Figure S3: IVA DWV-A contig sequences of the DWV inocula and DWV-inoculated pupae

Figure S4: IVA DWV-B contig sequences of the DWV inocula and DWV-inoculated pupae

Figure S5: Alignment of contigs generated by IVA assembly software using NGS datasets

Figure S6: Phylogeny of contigs generated by IVA assembly software using NGS datasets

Figure S7: Titres of DWV-A and DWV-B per honey bee pupa at days 0 and 3 post inoculation

Figure S8: Survival and wing deformities in treatment groups split by colony of origin

Figure S9: Viral titres per bee in eclosing honey bee adults inoculated with DWV-A and DWV-B

Table S1: Contents of NGS reads of DWV inocula

Table S2: Contents of NGS reads of eclosing adult hosts

Table S3: Comparison of published DWV sequences with our NGS libraries

Figure S1. Read coverage of the experimental inocula across the DWV genome (5′ → 3′). Genome-wide coverage of the DWV-A (a) and DWV-B (b) reads in the DWV-A and DWV-B inocula, respectively. The first and last 200 bp were excluded.

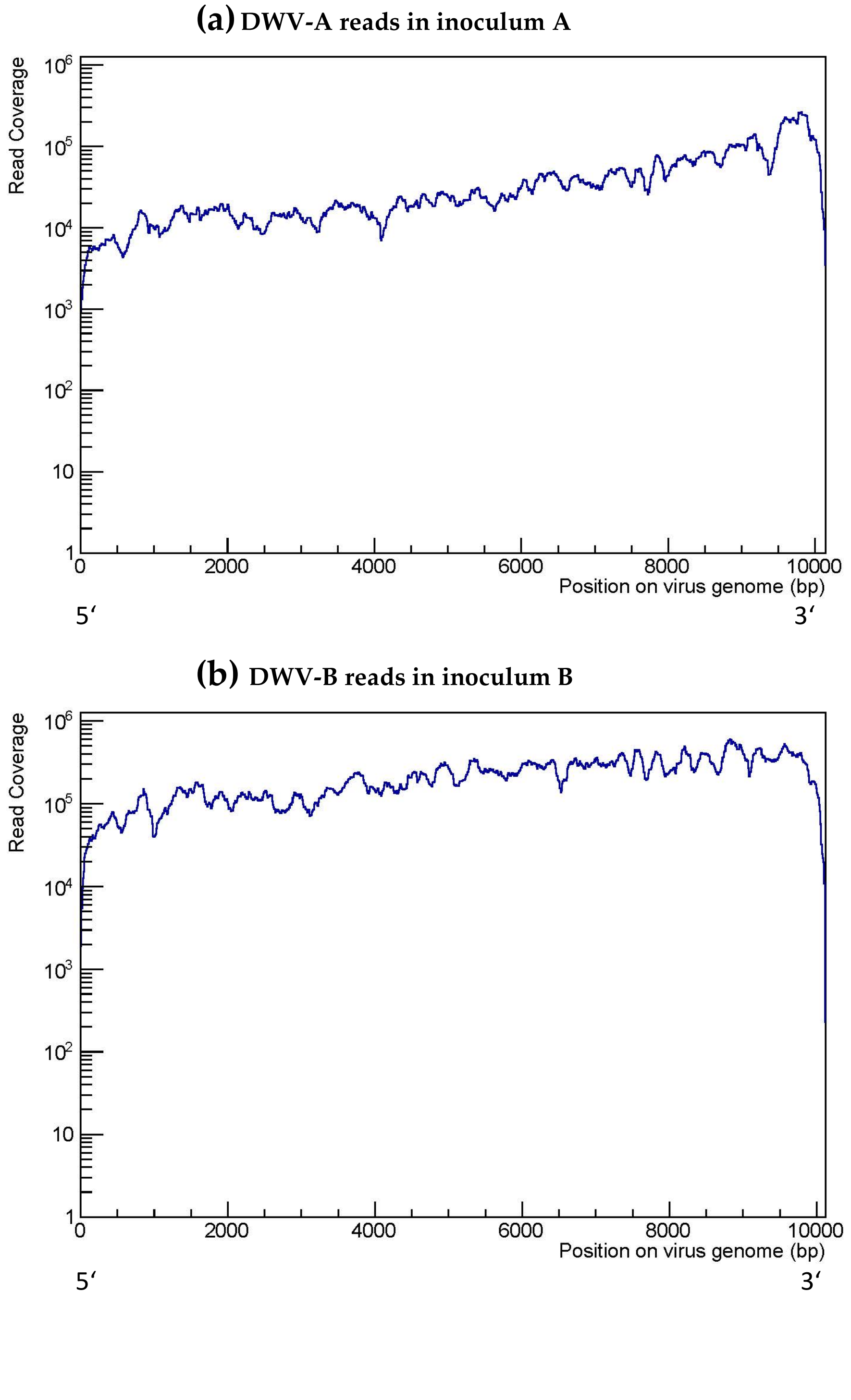

Figure S2. Genetic variability of the experimental inocula across the DWV genome (5′ → 3′). (a) Average genome-wide variability of DWV-A in the DWV-A inoculum and (b) DWV-B in the DWV-B inoculum. These represent nucleotide mismatches (SNPs) between DWV-A or -B reads when matched against their respective genome sequences (Figures S3, S4). Mutational variation around each isolate (DWV-A or -B) in inocula was calculated inside of non-overlapping 100 base pair windows using a custom script and was <0.5% whereas sequence divergence between isolates was >15% (Table S3), indicating that the two viral genotypes do not form an interconnected mutant cloud. The first and last 200 bp were excluded.

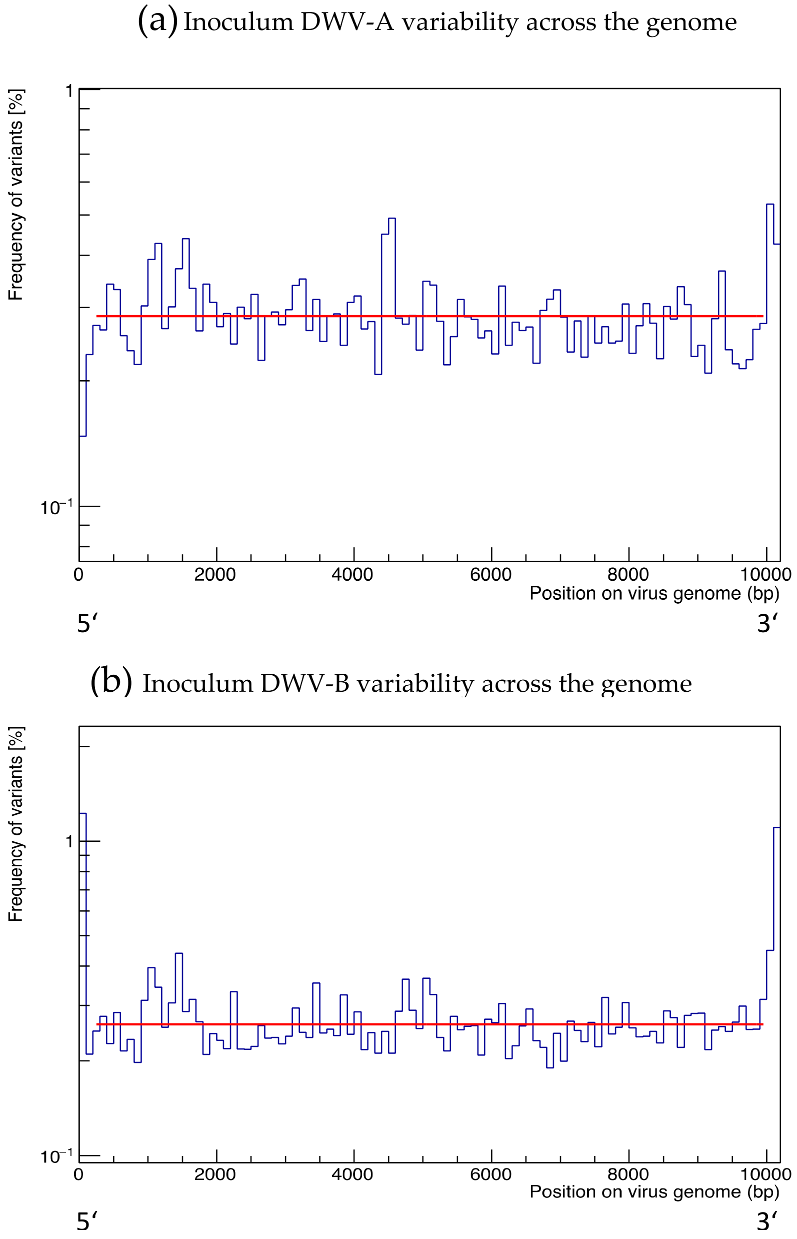

Figure S3. Full genome contig sequences of the DWV-A inoculum and DWV-A derived from a white-eyed host pupa experimentally inoculated with the DWV-A inoculum as it eclosed as an adult (individual pupal code: D4-DWV-A), as well as two partial DWV-A contigs from the DWV-B inoculum, as generated by IVA *de novo* assemblies of all DWV NGS reads.

>Inoculum-DWV-A; IVA full-genome DWV-A contig sequence
(Row 3 of Figure S5, code: DWV-A_contig.00001-Inoculum-A)

```
ATGGGAGGCGATTTATGCCTTCCATAGCGAATTACGGTGCAACTAACAATTTTAGATAGTAGCCATGAACAAACATTATA
GTAGCTCACTACGTATTGATCATTTTTATAATGACTTGCGTAGCATGAAGCGCATGCTTGTAGTTGTAACTATGTTACTT
TACAAGTTGGAGTTTACTATCTTGGATTATGAATATGTGCACTTAGTGTCTGTATTTATAGTCGTTTGTGGTTCAAGGTT
TTGTGTTAGTAGTACACTTATGTATGAATGTATCGTTAGTATGAATGTTATAGAATGACAATATCGAAAGAAAAATTTTT
ATAAAATACAAAAATATTGTTTTTATTATTTCGATATGGTGTTTTATAGAGTAGATTGCCATGTGACCGCTCATAGAAGT
CCATTATGGTTTATCAATCGAAGTTGAATGTATTTATAAGGATATTATACTTAATTAGTAATATTAGTAGTCCGTAACTA
TTATCATCCTTTTTCAGTTTGATGTGATAATAGACCACTGCAGTATCGAGTAGAGTTTCGAATGCGTAGTGCAATAGTAT
AATCACTGTCACCGACCATCTATTGTAATGATAGATCTGTCGGAAACCATTATTTATGAAGTGACTAGCAATCATGGATT
AAATTAGATGGTATTCTAATTTAGAGGTGATTCGGCGCTGCGGTGCGACTGAAACTTCTAAGTTAGCATGTCAGATTATA
TTATGAATGCGTTAGTAGTAATTTCTGCGATAGAGCTGGGACCCCTCAGTCTCTCAGGTATTGTACGAGGCGAAAGTGTG
AAAGTTTTGTATGTGTTTTTATATGTACGACTGTATCGGGAATTCCTTTAGCAAGAATCCTTTTAATACAGTATAATCTG
TGCTACGGTACGTTACGTTCGCAGGGCACCCGTTAATGTCTCATAGCCCAGACGATGGCGGATGGAAAGACATCATATTT
TATTTTAATGCTGTCTTTATTGCTGATTTATTTTGCTGTTTTTATTTGCTATTTTATATTTGCTAATTTTCATTATTGCG
AAATATATTACATTGCTATTTTTATTATTTACGCTAGATTCAATTTTATTTTTGTTATATTTTCAATTTAATTTTGATTT
CGAAGGTAAATATATATAATTAATTATTTAAAATGGCCTTTAGTTGTGGAACTCTTTCTTACTCTGCCGTCGCCCAAGCT
CCGTCTGTTGCCCATGCACCTCGTACATGGGAAGTTGATGAAGCTAGGCGGCGCCGAGTCATTAAACGTTTGGCGCTGGA
GCAAGAACGTATTCGTAACGTTCTTGACGTTGACGTCTATAACCAGGCGACATGGGAACAGGAGGACGCGCGCGATAACG
AGTTCCTAATGGAACAATTAAACAATTTATATACTATTTATTCGATCGCTGAACGTTGTACGCGTCGGCCTATTAAAGAG
TACTCTCCTATATCAGTTTCGAATAGGTTTGCTCCACTGGAATCCCTCAAGGTCGAGGTTGGTCAAGAAGCAAGCGAATG
TATATTTAAGAAACCTAAATATACGCGCGTTTGCAAGAAAGTGAAGCGTGTTGCAACTCGCTTCGTTTGTGAAAAAGTTG
TTCGTCCTATGTGTTCTAGATCCCCTATGCTATTATTTAAGCTTAAGAAAGTTATTTATGATTTGCATTTATATAGATTA
AGAAAACAGATTAGGATGTTAAGACGTCAAAAACAGCGCGATTATGAGTTAGAGTGTGTCACTAATCTGTTACAATTATC
GGATCCGGTGCAGGCAAAACCAGAGATGGATAACCCTAATCCAGGACCTGATGGCGAGGGTGAAGTTGAATTAGAAAAGG
ATAGTAATGTTGTTTTAACAACTCAGCGAGATCCTAGTACATCTATTCCAGCGCCGGTGAGCGTAAAATGGAGTAGATGG
ACTAGTAATGACGTAGTAGATGATTACGCCACAATTACATCTCGATGGTACCAGATTGCTGAATTTGTTTGGTCGAAGGA
TGATCCATTTGATAAGGAGTTAGCACGTTTAATTTTGCCTCGTGCTTTGTTATCTAGTATAGAGGCTAATTCTGACGCTA
TATGTGATGTGCCTAATACTATCCCATTTAAGGTACACGCATATTGGCGAGGCGATATGGAAGTTAGAGTTCAAATTAAT
TCAAATAAATTCCAAGTTGGTCAATTACAAGCTACTTGGTATTATTCGGATCATGAGAATTTGAATATATCGTCTAAGAG
AAGCGTTTATGGATTTTCACAAATGGATCATGCTTTGATTAGTGCGTCAGCAAGTAATGAAGCAAAATTAGTTATTCCAT
TTAAGCATGTTTATCCATTTTTACCGACAAGAATTGTGCCAGATTGGACTACTGGCATTTTAGATATGGGTGCTTTGAAC
ATTCGTGTAATTGCTCCCTTACGGATGAGTGCTACTGGTCCAACTACCTGTAATGTCGTCGTGTTTATTAAATTAAATAA
CAGCGAGTTTACAGGGACTTCTTCTGGTAAGTTTTATGCGAGCCAAATCAGGGCAAAACCTGAGATGGATCGTATATTAA
ATTTAGCAGAGGGATTGTTGAATAATACGATTGGTGGTAATAATATGGATAATCCTTCTTATCAACAATCTCCTCGTCAT
TTTGTCCCGACTGGTATGCACAGCTTAGCTTTAGGTACTAATTTAGTTGAACCATTACATGCATTACGTTTGGATGCAGC
CGGTACGACGCAACATCCTGTAGGTTGTGCTCCTGATGAAGATATGACTGTATCTTCCATTGCATCTCGATATGGACTAA
TTAGACGGGTACAATGGAAGAAAGATCATGCTAAAGGATCACTTTTGCTACAATTAGATGCTGATCCATTTGTGGAGCAA
AGAATTGAAGGTACGAATCCAATATCTTTGTATTGGTTCGCACCCGTGGGTGTAGTATCTAGTATGTTTATGCAATGGCG
TGGTTCATTAGAATATAGGTTTGATATTATAGCATCCCAATTTCATACTGGTAGGTTAATTGTAGGTTATGTGCCCGGTT
TGACAGCATCTTTGCAACTTCAAATGGACTATATGAAATTGAAGTCATCGAGTTATGTAGTATTTGATTTACAAGAAAGT
AATAGCTTCACTTTTGAGGTGCCATATGTTTCATATAGACCATGGTGGGTTCGTAAATATGGTGGTAATTATTTACCCTC
GTCAACTGATGCTCCTAGTACATTATTTATGTATGTGCAGGTTCCGTTGATACCTATGGAAGCTGTTTCAGATACTATTG
ATATCAATGTGTACGTACGGGGCGGTAGTTCATTTGAAGTTTGTGTTCCGGTCCAACCTAGTTTAGGTTTGAATTGGAAT
ACAGACTTTATTTTACGTAACGACGAAGAATACAGGGCTAAGACAGGTTATGCACCATATTATGCTGGAGTGTGGCATAG
CTTCAATAATAGTAATTCTCTTGTTTTTAGGTGGGGATCTGCTTCTGACCAAATTGCTCAGTGGCCAACAATTTCAGTAC
CCAGAGGTGAGCTAGCTTTCTTACGAATTAAGGATGGAAAGCAAGCTGCTGTAGGAACTCAACCTTGGCGTACGATGGTT
GTTTGGCCTTCTGGTCATGGTTATAATATTGGTATACCTACGTATAATGCTGAACGAGCTCGCCAGCTTGCACAACACTT
ATATGGTGGTGGATCATTAACTGATGAAAAGGCCAAACAATTATTTGTTCCTGCTAATCAACAAGGACCTGGTAAGGTAA
GTAATGGAAATCCGGTATGGGAAGTCATGCGCGCACCATTGGCAACACAGCGTGCGCATATTCAAGATTTTGAATTTATT
GAAGCTATTCCAGAAGGAGAGGAGTCTCGTAATACTACAGTCTTAGATACGACCACTACTTTACAGTCGAGTGGATTTGG
TCGCGCCTTCTTTGGTGAAGCTTTTAATGATCTTAAAACGTTAATGCGACGATATCAACTATATGGTCAATTATTATTGT
```

CCGTTACTACGGATAAGGATATTGATCATTGTATGTTTACCTTCCCTTGTTTACCACAAGGGTTAGCGTTAGACATTGGT
TCTGCTGGCTCTCCACATGAAATCTTTAATAGATGTCGTGATGGTATTATACCATTAATTGCATCTGGATATAGATTTTA
TAGAGGAGATTTGCGTTATAAGATTGTTTTTCCAAGTAATGTTAATAGCAATATTTGGGTACAACATCGACCGGATCGTA
GACTGGAAGGATGGTCCGCGGCTAAGATTGTAAATTGTGATGCTGTGTCTACTGGTCAAGGGGTGTATAATCATGGTTAT
GCTAGTCACATTCAAATTACGCGTGTAAATAATGTTATAGAATTGGAAGTTCCATTTTATAATGCTACTTGTTATAACTA
TTTACAGGCGTTTAATGCGTCTAGCGCTGCATCTAGTTACGCAGTATCTTTAGGGGAAATATCGGTTGGTTTTCAAGCTA
CAAGTGATGATATTGCATCTATTGTTAATAAACCTGTTACTATTTATTATAGTATTGGAGATGGTATGCAATTTTCTCAG
TGGGTCGGATATCAACCGATGATGATTTTAGACCAGCTTCCTGCACCAGTAGTAAGGGCCGTGCCTGAGGGCCCTATTGC
GAAGATTAAAAACTTCTTTCATCAAACAGCCGACGAAGTTAGAGAAGCTCAGGCAGCAAAGATGCGTGAAGATATGGGTA
TGGTTGTCCAAGATGTTATTGGAGAACTTAGCCAGGCCATACCGGATCTTCAACAACCGGAGGTTCAAGCAAATGTCTTC
TCACTGGTGTCTCAGTTAGTGCATGCTATTATAGGTACTAGTTTGAAGACAGTTGCTTGGGCGATTGTTTCGATTTTTGT
GACTTTAGGACTAATTGGACGCGAAATGATGCATTCAGTTATTACTGTAGTCAAGCGGTTACTTGAGAAATATCACTTGG
CGACGCAACCCCAGGAATCCGCCAGTTCAAGTACTGTTATATCTGCCGTTCCAGAAGCTCCCAATGCTGAAGCGGAGGAG
GCAAGTGCTTGGGTATCCATTATTTATAATGGTGTGTGTAATATGCTTAATGTGGCTGCTCAAAAACCGAAACAATTTAA
AGATTGGGTAAAATTAGCTACTGTAGATTTTAGTAATAATTGTAGAGGTAGTAACCAGGTATTTGTATTTTTCAAGAATA
CATTTGAAGTGTTGAAGAAAATGTGGGGTTATGTATTTTGTCAGAGTAATCCTGCAGCGCGTTTGTTGAAAGCTGTGAAT
GACGAGCCTGAGATTTTGAAAGCATGGGTGAAGGAATGTCTATATTTGGATGATCCCAAATTTAGAATGCGTCGAGCGCA
TGATCAAGAGTATATCGAGAGAGTGTTTGCGGCACATTCATATGGACAAATTCTGCTACATGATTTAACTGCTGAAATGA
ATCAATCACGAAATTTGAGTGTGTTTACACGTGTGTATGATCAAATTTCAAAATTGAAGACCGATCTTATGGAAATGGGA
TCGAATCCATATATAAGGCGTGAATGTTTTACGATATGCATGTGTGGTGCATCTGGAATTGGAAAATCATATTTGACCGA
TTCTTTATGCAGCGAGCTCTTACGTGCGAGTCGTACTCCTGTGACAACAGGCATAAAATGTGTTGTTAATCCATTATCTG
ATTATTGGGATCAATGTGATTTTCAGCCTGTTTTGTGCGTTGACGATATGTGGAGTGTTGAAACATCTACTACGCTTGAT
AAGCAGTTGAATATGCTTTTCCAGGTCCATTCCCCTATCGTGCTTTCTCCTCCTAAAGCTGATTTAGAAGGTAAGAAAAT
GCGATATAATCCAGAAATATTCATATACAATACGAATAAACCTTTCCCGAGGTTTGATCGTATTGCTATGGAAGCTATTT
ATCGGCGTAGAAATGTTTTGATTGAATGTAAAGCGAGTGAAGAGAAGAAGCGAGGATGTAAGCATTGTGAGAATGATATT
CCTATTGCTGAATGCAGTCCTAAGATGTTGAAAGATTTTCATCATATTAAATTTAGGTATGCACATGATGTATGCAATTC
CGAAACCACATGGTCTGAATGGATGACGTATAATGAATTTCTTGAATGGATAACTCCTGTGTATATGGCTAACCGTCGTA
AGGCGAATGAATCGTTTAAGATGCGTGTGGATGAAATGCAAATGTTACGTATGGATGAACCATTAGAAGGTGATAATATT
CTCAATAAGTATGTTGAAGTTAATCAGCGCTTAGTGGAGGAAATGAAGGCATTTAAGGAGCGTACACTATGGTCAGATTT
ACATCGCGTAGGTGCGGAAATTAGTGCGTCAGTTAAGAAAGCTTTGCCAACCATTTCCATAACCGAAAAATTACCACATT
GGACTGTTCAATGTGGTATTGCTAAACCTGAGATGGATCATGCTTATGAGGTTATGAGTTCGTATGCAGCTGGAATGAAT
GCAGAGATTGAAGCGCATGAACAAGTTCGGCGTTCATCAGTGGAATGTCAATATGCAGAACCTCAAGCTCCAAGAAATCC
TGATGATGAAGGACCAACCATAGATGAAGAACTTATGGGCGACACTGAATTTACATCACAGGCTCTAGAACGTCTTGTGG
ATGAAGGTTATATAACTGGAAAACAGAAGAAATATATAGCTACGTGGTGTAGTAAGCGTCGTGAACATATTGCTGACTTT
GATCTTGTGTGGACTGATAATTTGCGTGTGTTAAGTGCGTATGTGCATGAACGTTCGTCTTCAACTCGGCTTTCTACGGA
TGACGTCAAGTTATATAAAACAATTAGCATGTTGCATCAAAAATATGACACCACAGAGTGTGCTAAATGTCAACATTGGT
ATGCTCCGTTGACTGATATCTATGTTGATGACAAGAAATTGTTTTGGTGTCAGAAAGAGAAAAAGACACTTATTGATGTC
CGAAAATTGTCGAAAGAAGATGTGACTGTTCAATCAAAATTGATTAATTTATCTGTTCCTTGTGGTGAAGTGTGTATGTT
GCATTCAAAATATTTCAATTATCTTTTCCATAAAGCATGGTTGTTTGAGAACCCAACTTGGCGCCTAATATATAATGGTA
CCAAGAAGGGTATGCCTGAGTACTTTATGAATTGTGTGGATGAGATTTCATTAGATTCCAAATTTGGTAAAGTGAAAGTG
TGGTTGCAAGCGATCATTGATAAGTATTTAACTCGTCCCGTGAAAATGATTCGTGATTTTCTTTTCAAGTGGTGGCCGCA
AGTTGCGTATGTGTTGAGCTTGCTAGGTATAATTGGTATAACTGCGTATGAAATGAGAAATCCGAAACCAACTTCTGAGG
AATTAGTTGATCATTATGTAAATAGGCACTGTAGTTCTGATTTTTGGTCACCAGGAATGGCAACACCTCAAGGATTGAAA
TATAGTGAAGCAGTAACAGCAAGGGCACCTCGAATTCATAGATTGCCAGTTACTACTAAGCCTCAGGGATCAACACAACA
AGTAGACGCTGCTGTGAATAAAATTTTACAGAACATGGTTTACATTGGTGTTGTTTTTCCAAAAGTGCCTGGTAGTAAGT
GGCGAGACATTAATTTTAGGTGTCTTATGCTTCATAATAGGCAATGTTTAATGTTGAGGCATTATATTGAGTCAACTGCC
GCCTTTCCTGAGGGAACTAAGTACTATTTTAAGTATATTCATAATCAAGAGACTAGAATGTCTGGGGATATTTCTGGTAT
TGAAATTGATTTGTTGAATTTACCTAGATTGTATTATGGTGGTCTCGCGGGAGAGGAGTCATTTGATAGCAATATCGTGC
TTGTGACTATGCCTAATCGTATTCCTGAGTGTAAGAGCATTATTAAGTTTATAGCGTCACATAATGAACACATACGTGCT
CAGAATGATGGAGTGTTAGTAACTGGCGACCATACTCAGCTATTGGCTTTCGAGAATAATAATAAAACTCCAATAAGTAT
CAACGCTGATGGTTTGTATGAGGTTATACTTCAAGGAGTATATACTTATCCATACCATGGCGACGGTGTTTGTGGTTCCA
TATTGTTATCTCGGAATTTACAACGGCCAATTATAGGTATCCATGTTGCTGGTACTGAAGGATTGCATGGCTTTGGAGTT
GCTGAACCACTTGTACATGAAATGTTCACTGGTAAAGCAATTGAGAGTGAGAGAGAACCGTATGATCGTGTGTATGAACT
TCCGTTGCGTGAATTAGATGAATCTGATATTGGTTTAGATACTGATTTATATCCGATTGGTAGAGTGGATGCAAAGCTAG
CTCATGCTCAAAGCCCTTCTACTGGGATTAAAAAGACGCTTATCCATGGAACATTTGACGTAAGGACTGAACCAAATCCG
ATGTCATCACGTGATCCAAGAATAGCGCCGCATGATCCTTTGAAGTTAGGGTGTGAAAAGCATGGAATGCCCTGTTCACC
GTTTAATAGGAAACATTTGGAATTAGCGACAAATCATTTGAAAGAAAAACTAATTTCAGTAGTTAAACCAATAAATGGAT
GCAAGATTAGAAGTTTGCAAGATGCTGTATGTGGTGTGCCTGGTTTAGATGGGTTTGATTCGATATCTTGGAATACTAGT
GCTGGCTTTCCTTTGTCTTCATTAAAGCCACCTGGAACATCAGGTAAGCGATGGTTGTTTGACATTGAGCTACAAGACTC
GGGATGTTATCTCTTGCGTGGAATGCGTCCCGAACTTGAGATTCAATTATCAACGACACAGTTAATGAGGAAAAAGGGAA
TAAAACCTCACACTATATTCACGGATTGTTTGAAAGATACTTGTTTGCCTGTGGAAAAATGTAGAATACCTGGTAAGACT
AGAATATTTAGTATAAGTCCGGTACAATTTACTATACCGTTTAGACAGTATTACTTAGATTTTATGGCATCCTATCGAGC
TGCACGCCTTAATGCTGAGCATGGTATCGGTATTGATGTTAACAGCTTAGAATGGACAAATTTGGCAACGAGTTTGTCGA

AGTATGGCACTCATATCGTGACGGGTGACTATAAGAATTTTGGCCCTGGATTAGATTCCGATGTTGCAGCTTCGGCGTTT
GAAATTATTATCGACTGGGTATTACATTATACTGAAGAAGATAATAAAGACGAAATGAAGCGAGTAATGTGGACCATGGC
GCAAGAGATTCTAGCGCCTAGTCATCTATGTCGTGATTTAGTGTACCGAGTACCTTGTGGAATTCCATCAGGTTCTCCGA
TAACGGACATTTTGAATACAATTTCAAATTGTCTGTTAATTAGGTTAGCTTGGCTAGGTATTACTGATTTGCCTTTATCC
GAGTTCTCTCAAAATGTTGTTCTTGTTTGTTATGGTGATGATCTTATCATGAATGTTAGTGATAACATGATTGACAAATT
TAATGCTGTGACAATAGGGAAATTCTTTTCACAATATAAGATGGAATTTACGGATCAGGACAAATCAGGAAATACTGTGA
AGTGGCGGACGTTACAGACTGCTACTTTCTTGAAGCATGGGTTTTTAAAACATCCGACTAGACCTGTGTTTCTGGCTAAC
CTAGACAAGGTTTCGGTAGAAGGAACGACGAATTGGACCCATGCTCGGGGATTGGGTCGTCGTGCAGCAACCATAGAGAA
TGCTAAACAAGCGCTAGAGTTAGCATTCGGATGGGGTCCAGAATACTTTAACTATGTCAGAAATACTATTAAAATGGCTT
TTGACAAGTTGGGTATTTATGAAGACCTTATCACATGGGAAGAAATGGATGTTAGATGTTATGCTAGCGCGTAGTATTTA
ATTTTGAATACTCATTAGTTTTAATTTTATTTTAGGTTATTGGAATTGAGGGAAGTACCACCCCCCAAGACCTTCGTTTT
AAATCTACTAGAAGGAGTGAACCTATATATAAGAGTCTAAAGACAGAGTGGATTAGACCATCATCTTTAGCTTATATATG
GGGAAGGTTGAGTTGCCTCTAAAGACTCAGCTCCATAGTAGAGTAGTTTTAATTACGATTAAAGTGGTACTCTAGGTTAG
GTGTTACTCGCGTATTATCAACTAGTGGTAATGCGTCCTAATCTTAGTATAGTTTTAACCATAATAGTAAAAAAAAAAAA
AAAAAAAAAAAAAAAAAAAAAAAAAAAAAAAAAAAAAAAAAAAAAAAAAAAAAAAAAAAAAAAAAAAAAAAAAAAAAAAA
GAAAAAAAAAAAAAAAAAAAAAAAAAAAAA

> Code of pupa: D4-DWV-A; IVA full genome DWV-A contig sequence
(Row 4 of Figure S5, code: DWV-A_contig.00001-D4-A)
TGGCGATTTATGCCTTCCATAGCGAATTACGGTGCAACTAACAATTTTAGATAGTAGCCATGAACAAACATTATAGTAGC
TCACTACGTATTGATCATTTTTATAATGACTTGCGTAGCATGAAGCGCATGCTTGTAGTTGTAACTATGTTACTTTACAA
GTTGGAGTTTACTATCTTGGATTATGAATATGTGCACTTAGTGTCTGTATTTATAGTCGTTTGTGGTTCAAGGTTTTGTG
TTAGTAGTACACTTATGTATGAATGTATCGTTAGTATGAATGTTATAGAATGACAATATCGAAAGAAAAATTTTTATAAA
ATACAAAAATATTGTTTTTATTATTTCGATATGGTGTTTTATAGAGTAGATTGCCATGTGACCGCTCATAGAAGTCCATT
ATGGTTTATCAATCGAAGTTGAATGTATTTATAAGGATATTATACTTAATTAGTAATATTAGTAGTCCGTAACTATTATC
ATCCTTTTTCAGTTTGATGTGATAATAGACCACTGCAGTATCGAGTAGAGTTTCGAATGCGTAGTGCAATAGTATAATCA
CTGTCACCGACCATCTATTGTAATGATAGATCTGTCGGAAACCATTATTTATGAAGTGACTAGCAATCATGGATTAAATT
AGATGGTATTCTAATTTAGAGGTGATTCGGCGCTGCGGTGCGACTGAAACTTCTAAGTTAGCATGTCAGATTATATTATG
AATGCGTTAGTAGTAATTTCTGCGATAGAGCTGGGACCCCTCAGTCTCTCAGGTATTGTACGAGGCGAAAGTGTGAAAGT
TTTGTATGTGTTTTTATATGTACGACTGTATCGGGAATTCCTTTAGCAAGAATCCTTTTAATACAGTATAATCTGTGCTA
CGGTACGTTACGTTCGCAGGGCACCCGTTAATGTCTCATAGCCCAGACGATGGCGGATGGAAAGACATCATATTTTATTT
TAATGCTGTCTTTATTGCTGATTTATTTTGCTGTTTTTATTTGCTATTTTATATTTGCTAATTTTCATTATTGCGAAATA
TATTACATTGCTATTTTTATTATTTACGCTAGATTCAATTTTATTTTTGTTATATTTTCAATTTAATTTTGATTTCGAAG
GTAAATATATATAATTAATTATTTAAAATGGCCTTTAGTTGTGGAACTCTTTCTTACTCTGCCGTCGCCCAAGCTCCGTC
TGTTGCCCATGCACCTCGTACATGGGAAGTTGATGAAGCTAGGCGGCGCCGAGTCATTAAACGTTTGGCGCTGGAGCAAG
AACGTATTCGTAACGTTCTTGACGTTGACGTCTATAACCAGGCGACATGGGAACAGGAGGACGCGCGCGATAACGAGTTC
CTAATGGAACAATTAAACAATTTATATACTATTTATTCGATCGCTGAACGTTGTACGCGTCGGCCTATTAAAGAGTACTC
TCCTATATCAGTTTCGAATAGGTTTGCTCCACTGGAATCCCTCAAGGTCGAGGTTGGTCAAGAAGCAAGCGAATGTATAT
TTAAGAAACCTAAATATACGCGCGTTTGCAAGAAAGTGAAGCGTGTTGCAACTCGCTTCGTTTGTGAAAAAGTTGTTCGT
CCTATGTGTTCTAGATCCCCTATGCTATTATTTAAGCTTAAGAAAGTTATTTATGATTTGCATTTATATAGATTAAGAAA
ACAGATTAGGATGTTAAGACGTCAAAAACAGCGCGATTATGAGTTAGAGTGTGTCACTAATCTGTTACAATTATCGGATC
CGGTGCAGGCAAAACCAGAGATGGATAACCCTAATCCAGGACCTGATGGCGAGGGTGAAGTTGAATTAGAAAAGGATAGT
AATGTTGTTTTAACAACTCAGCGAGATCCTAGTACATCTATTCCAGCGCCGGTGAGCGTAAAATGGAGTAGATGGACTAG
TAATGACGTAGTAGATGATTACGCCACAATTACATCTCGATGGTACCAGATTGCTGAATTTGTTTGGTCGAAGGATGATC
CATTTGATAAGGAGTTAGCACGTTTAATTTTGCCTCGTGCTTTGTTATCTAGTATAGAGGCTAATTCTGACGCTATATGT
GATGTGCCTAATACTATCCCATTTAAGGTACACGCATATTGGCGAGGCGATATGGAAGTTAGAGTTCAAATTAATTCAAA
TAAATTCCAAGTTGGTCAATTACAAGCTACTTGGTATTATTCGGATCATGAGAATTTGAATATATCGTCTAAGAGAAGCG
TTTATGGATTTTCACAAATGGATCATGCTTTGATTAGTGCGTCAGCAAGTAATGAAGCAAAATTAGTTATTCCATTTAAG
CATGTTTATCCATTTTTACCGACAAGAATTGTGCCAGATTGGACTACTGGCATTTTAGATATGGGTGCTTTGAACATTCG
TGTAATTGCTCCCTTACGGATGAGTGCTACTGGTCCAACTACCTGTAATGTCGTCGTGTTTATTAAATTAAATAACAGCG
AGTTTACAGGGACTTCTTCTGGTAAGTTTTATGCGAGCCAAATCAGGGCAAAACCTGAGATGGATCGTATATTAAATTTA
GCAGAGGGATTGTTGAATAATACGATTGGTGGTAATAATATGGATAATCCTTCTTATCAACAATCTCCTCGTCATTTTGT
CCCGACTGGTATGCACAGCTTAGCTTTAGGTACTAATTTAGTTGAACCATTACATGCATTACGTTTGGATGCAGCCGGTA
CGACGCAACATCCTGTAGGTTGTGCTCCTGATGAAGATATGACTGTATCTTCCATTGCATCTCGATATGGACTAATTAGA
CGGGTACAATGGAAGAAAGATCATGCTAAAGGATCACTTTTGCTACAATTAGATGCTGATCCATTTGTGGAGCAAAGAAT
TGAAGGTACGAATCCAATATCTTTGTATTGGTTCGCACCCGTGGGTGTAGTATCTAGTATGTTTATGCAATGGCGTGGTT
CATTAGAATATAGGTTTGATATTATAGCATCCCAATTTCATACTGGTAGGTTAATTGTAGGTTATGTGCCCGGTTTGACA
GCATCTTTGCAACTTCAAATGGACTATATGAAATTGAAGTCATCGAGTTATGTAGTATTTGATTTACAAGAAAGTAATAG
CTTCACTTTTGAGGTGCCATATGTTTCATATAGACCATGGTGGGTTCGTAAATATGGTGGTAATTATTTACCCTCGTCAA
CTGATGCTCCTAGTACATTATTTATGTATGTGCAGGTTCCGTTGATACCTATGGAAGCTGTTTCAGATACTATTGATATC
AATGTGTACGTACGGGGCGGTAGTTCATTTGAAGTTTGTGTTCCGGTCCAACCTAGTTTAGGTTTGAATTGGAATACAGA
CTTTATTTTACGTAACGACGAAGAATACAGGGCTAAGACAGGTTATGCACCATATTATGCTGGAGTGTGGCATAGCTTCA

ATAATAGTAATTCTCTTGTTTTTAGGTGGGGATCTGCTTCTGACCAAATTGCTCAGTGGCCAACAATTTCAGTACCCAGA
GGTGAGCTAGCTTTCTTACGAATTAAGGATGGAAAGCAAGCTGCTGTAGGAACTCAACCTTGGCGTACGATGGTTGTTTG
GCCTTCTGGTCATGGTTATAATATTGGTATACCTACGTATAATGCTGAACGAGCTCGCCAGCTTGCACAACACTTATATG
GTGGTGGATCATTAACTGATGAAAAGGCCAAACAATTATTTGTTCCTGCTAATCAACAAGGACCTGGTAAGGTAAGTAAT
GGAAATCCGGTATGGGAAGTCATGCGCGCACCATTGGCAACACAGCGTGCGCATATTCAAGATTTTGAATTTATTGAAGC
TATTCCAGAAGGAGAGGAGTCTCGTAATACTACAGTCTTAGATACGACCACTACTTTACAGTCGAGTGGATTTGGTCGCG
CCTTCTTTGGTGAAGCTTTTAATGATCTTAAAACGTTAATGCGACGATATCAACTATATGGTCAATTATTATTGTCCGTT
ACTACGGATAAGGATATTGATCATTGTATGTTTACCTTCCCTTGTTTACCACAAGGGTTAGCGTTAGACATTGGTTCTGC
TGGCTCTCCACATGAAATCTTTAATAGATGTCGTGATGGTATTATACCATTAATTGCATCTGGATATAGATTTTATAGAG
GAGATTTGCGTTATAAGATTGTTTTTCCAAGTAATGTTAATAGCAATATTTGGGTACAACATCGACCGGATCGTAGACTG
GAAGGATGGTCCGCGGCTAAGATTGTAAATTGTGATGCTGTGTCTACTGGTCAAGGGGTGTATAATCATGGTTATGCTAG
TCACATTCAAATTACGCGTGTAAATAATGTTATAGAATTGGAAGTTCCATTTTATAATGCTACTTGTTATAACTATTTAC
AGGCGTTTAATGCGTCTAGCGCTGCATCTAGTTACGCAGTATCTTTAGGGGAAATATCGGTTGGTTTTCAAGCTACAAGT
GATGATATTGCATCTATTGTTAATAAACCTGTTACTATTTATTATAGTATTGGAGATGGTATGCAATTTTCTCAGTGGGT
CGGATATCAACCGATGATGATTTTAGACCAGCTTCCTGCACCAGTAGTAAGGGCCGTGCCTGAGGGCCCTATTGCGAAGA
TTAAAAACTTCTTTCATCAAACAGCCGACGAAGTTAGAGAAGCTCAGGCAGCAAAGATGCGTGAAGATATGGGTATGGTT
GTCCAAGATGTTATTGGAGAACTTAGCCAGGCCATACCGGATCTTCAACAACCGGAGGTTCAAGCAAATGTCTTCTCACT
GGTGTCTCAGTTAGTGCATGCTATTATAGGTACTAGTTTGAAGACAGTTGCTTGGGCGATTGTTTCGATTTTTGTGACTT
TAGGACTAATTGGACGCGAAATGATGCATTCAGTTATTACTGTAGTCAAGCGGTTACTTGAGAAATATCACTTGGCGACG
CAACCCCAGGAATCCGCCAGTTCAAGTACTGTTATATCTGCCGTTCCAGAAGCTCCCAATGCTGAAGCGGAGGAGGCAAG
TGCTTGGGTATCCATTATTTATAATGGTGTGTGTAATATGCTTAATGTGGCTGCTCAAAAACCGAAACAATTTAAAGATT
GGGTAAAATTAGCTACTGTAGATTTTAGTAATAATTGTAGAGGTAGTAACCAGGTATTTGTATTTTTCAAGAATACATTT
GAAGTGTTGAAGAAAATGTGGGGTTATGTATTTTGTCAGAGTAATCCTGCAGCGCGTTTGTTGAAAGCTGTGAATGACGA
GCCTGAGATTTTGAAAGCATGGGTGAAGGAATGTCTATATTTGGATGATCCCAAATTTAGAATGCGTCGAGCGCATGATC
AAGAGTATATCGAGAGAGTGTTTGCGGCACATTCATATGGACAAATTCTGCTACATGATTTAACTGCTGAAATGAATCAA
TCACGAAATTTGAGTGTGTTTACACGTGTGTATGATCAAATTTCAAAATTGAAGACCGATCTTATGGAAATGGGATCGAA
TCCATATATAAGGCGTGAATGTTTTACGATATGCATGTGTGGTGCATCTGGAATTGGAAAATCATATTTGACCGATTCTT
TATGCAGCGAGCTCTTACGTGCGAGTCGTACTCCTGTGACAACAGGCATAAAATGTGTTGTTAATCCATTATCTGATTAT
TGGGATCAATGTGATTTTCAGCCTGTTTTGTGCGTTGACGATATGTGGAGTGTTGAAACATCTACTACGCTTGATAAGCA
GTTGAATATGCTTTTCCAGGTCCATTCCCCTATCGTGCTTTCTCCTCCTAAAGCTGATTTAGAAGGTAAGAAAATGCGAT
ATAATCCAGAAATATTCATATACAATACGAATAAACCTTTCCCGAGGTTTGATCGTATTGCTATGGAAGCTATTTATCGG
CGTAGAAATGTTTTGATTGAATGTAAAGCGAGTGAAGAGAAGAAGCGAGGATGTAAGCATTGTGAGAATGATATTCCTAT
TGCTGAATGCAGTCCTAAGATGTTGAAAGATTTTCATCATATTAAATTTAGGTATGCACATGATGTATGCAATTCCGAAA
CCACATGGTCTGAATGGATGACGTATAATGAATTTCTTGAATGGATAACTCCTGTGTATATGGCTAACCGTCGTAAGGCG
AATGAATCGTTTAAGATGCGTGTGGATGAAATGCAAATGTTACGTATGGATGAACCATTAGAAGGTGATAATATTCTCAA
TAAGTATGTTGAAGTTAATCAGCGCTTAGTGGAGGAAATGAAGGCATTTAAGGAGCGTACACTATGGTCAGATTTACATC
GCGTAGGTGCGGAAATTAGTGCGTCAGTTAAGAAAGCTTTGCCAACCATTTCCATAACCGAAAAATTACCACATTGGACT
GTTCAATGTGGTATTGCTAAACCTGAGATGGATCATGCTTATGAGGTTATGAGTTCGTATGCAGCTGGAATGAATGCAGA
GATTGAAGCGCATGAACAAGTTCGGCGTTCATCAGTGGAATGTCAATATGCAGAACCTCAAGCTCCAAGAAATCCTGATG
ATGAAGGACCAACCATAGATGAAGAACTTATGGGCGACACTGAATTTACATCACAGGCTCTAGAACGTCTTGTGGATGAA
GGTTATATAACTGGAAAACAGAAGAAATATATAGCTACGTGGTGTAGTAAGCGTCGTGAACATATTGCTGACTTTGATCT
TGTGTGGACTGATAATTTGCGTGTGTTAAGTGCGTATGTGCATGAACGTTCGTCTTCAACTCGGCTTTCTACGGATGACG
TCAAGTTATATAAAACAATTAGCATGTTGCATCAAAAATATGACACCACAGAGTGTGCTAAATGTCAACATTGGTATGCT
CCGTTGACTGATATCTATGTTGATGACAAGAAATTGTTTTGGTGTCAGAAAGAGAAAAAGACACTTATTGATGTCCGAAA
ATTGTCGAAAGAAGATGTGACTGTTCAATCAAAATTGATTAATTTATCTGTTCCTTGTGGTGAAGTGTGTATGTTGCATT
CAAAATATTTCAATTATCTTTTCCATAAAGCATGGTTGTTTGAGAACCCAACTTGGCGCCTAATATATAATGGTACCAAG
AAGGGTATGCCTGAGTACTTTATGAATTGTGTGGATGAGATTTCATTAGATTCCAAATTTGGTAAAGTGAAAGTGTGGTT
GCAAGCGATCATTGATAAGTATTTAACTCGTCCCGTGAAAATGATTCGTGATTTTCTTTTCAAGTGGTGGCCGCAAGTTG
CGTATGTGTTGAGCTTGCTAGGTATAATTGGTATAACTGCGTATGAAATGAGAAATCCGAAACCAACTTCTGAGGAATTA
GTTGATCATTATGTAAATAGGCACTGTAGTTCTGATTTTTGGTCACCAGGAATGGCAACACCTCAAGGATTGAAATATAG
TGAAGCAGTAACAGCAAGGGCACCTCGAATTCATAGATTGCCAGTTACTACTAAGCCTCAGGGATCAACACAACAAGTAG
ACGCTGCTGTGAATAAAATTTTACAGAACATGGTTTACATTGGTGTTGTTTTTCCAAAAGTGCCTGGTAGTAAGTGGCGA
GACATTAATTTTAGGTGTCTTATGCTTCATAATAGGCAATGTTTAATGTTGAGGCATTATATTGAGTCAACTGCCGCCTT
TCCTGAGGGAACTAAGTACTATTTTAAGTATATTCATAATCAAGAGACTAGAATGTCTGGGGATATTTCTGGTATTGAAA
TTGATTTGTTGAATTTACCTAGATTGTATTATGGTGGTCTCGCGGGAGAGGAGTCATTTGATAGCAATATCGTGCTTGTG
ACTATGCCTAATCGTATTCCTGAGTGTAAGAGCATTATTAAGTTTATAGCGTCACATAATGAACACATACGTGCTCAGAA
TGATGGAGTGTTAGTAACTGGCGACCATACTCAGCTATTGGCTTTCGAGAATAATAATAAAACTCCAATAAGTATCAACG
CTGATGGTTTGTATGAGGTTATACTTCAAGGAGTATATACTTATCCATACCATGGCGACGGTGTTTGTGGTTCCATATTG
TTATCTCGGAATTTACAACGGCCAATTATAGGTATCCATGTTGCTGGTACTGAAGGATTGCATGGCTTTGGAGTTGCTGA
ACCACTTGTACATGAAATGTTCACTGGTAAAGCAATTGAGAGTGAGAGAGAACCGTATGATCGTGTGTATGAACTTCCGT
TGCGTGAATTAGATGAATCTGATATTGGTTTAGATACTGATTTATATCCGATTGGTAGAGTGGATGCAAAGCTAGCTCAT
GCTCAAAGCCCTTCTACTGGGATTAAAAAGACGCTTATCCATGGAACATTTGACGTAAGGACTGAACCAAATCCGATGTC
ATCACGTGATCCAAGAATAGCGCCGCATGATCCTTTGAAGTTAGGGTGTGAAAAGCATGGAATGCCCTGTTCACCGTTTA

ATAGGAAACATTTGGAATTAGCGACAAATCATTTGAAAGAAAAACTAATTTCAGTAGTTAAACCAATAAATGGATGCAAG
ATTAGAAGTTTGCAAGATGCTGTATGTGGTGTGCCTGGTTTAGATGGGTTTGATTCGATATCTTGGAATACTAGTGCTGG
CTTTCCTTTGTCTTCATTAAAGCCACCTGGAACATCAGGTAAGCGATGGTTGTTTGACATTGAGCTACAAGACTCGGGAT
GTTATCTCTTGCGTGGAATGCGTCCCGAACTTGAGATTCAATTATCAACGACACAGTTAATGAGGAAAAAGGGAATAAAA
CCTCACACTATATTCACGGATTGTTTGAAAGATACTTGTTTGCCTGTGGAAAAATGTAGAATACCTGGTAAGACTAGAAT
ATTTAGTATAAGTCCGGTACAATTTACTATACCGTTTAGACAGTATTACTTAGATTTTATGGCATCCTATCGAGCTGCAC
GCCTTAATGCTGAGCATGGTATCGGTATTGATGTTAACAGCTTAGAATGGACAAATTTGGCAACGAGTTTGTCGAAGTAT
GGCACTCATATCGTGACGGGTGACTATAAGAATTTTGGCCCTGGATTAGATTCCGATGTTGCAGCTTCGGCGTTTGAAAT
TATTATCGACTGGGTATTACATTATACTGAAGAAGATAATAAAGACGAAATGAAGCGAGTAATGTGGACCATGGCGCAAG
AGATTCTAGCGCCTAGTCATCTATGTCGTGATTTAGTGTACCGAGTACCTTGTGGAATTCCATCAGGTTCTCCGATAACG
GACATTTTGAATACAATTTCAAATTGTCTGTTAATTAGGTTAGCTTGGCTAGGTATTACTGATTTGCCTTTATCCGAGTT
CTCTCAAAATGTTGTTCTTGTTTGTTATGGTGATGATCTTATCATGAATGTTAGTGATAACATGATTGACAAATTTAATG
CTGTGACAATAGGGAAATTCTTTTCACAATATAAGATGGAATTTACGGATCAGGACAAATCAGGAAATACTGTGAAGTGG
CGGACGTTACAGACTGCTACTTTCTTGAAGCATGGGTTTTTAAAACATCCGACTAGACCTGTGTTTCTGGCAAACCTAGA
CAAGGTTTCGGTAGAAGGAACGACGAATTGGACCCATGCTCGGGGATTGGGTCGTCGTGCAGCAACCATAGAGAATGCTA
AACAAGCGCTAGAGTTAGCATTCGGATGGGGTCCAGAATACTTTAACTATGTCAGAAATACTATTAAAATGGCTTTTGAC
AAGTTGGGTATTTATGAAGACCTTATCACATGGGAAGAAATGGATGTTAGATGTTATGCTAGCGCGTAGTATTTAATTTT
GAATACTCATTAGTTTTAATTTTATTTTAGGTTATTGGAATTGAGGGAAGTACCACCCCCCAAGACCTTCGTTTTAAATC
TACTAGAAGGAGTGAACCTATATATAAGAGTCTAAAGACAGAGTGGATTAGACCATCATCTTTAGCTTATATATGGGGAA
GGTTGAGTTGCCTCTAAAGACTCAGCTCCATAGTAGAGTAGTTTTAATTACGATTAAAGTGGTACTCTAGGTTAGGTGTT
ACTCGCGTATTATCAACTAGTGGTAATGCGTCCTAATCTTAGTATAGTTTTAACCATAATAGTAAAAAAAAAAAAAAAAA
AAAAAAAAA

>Inoculum-DWV-B; IVA partial genome DWV-A contig sequence
(Row 5 of Figure S5, code: DWV-A_contig.00002-Inoculum-B)
AGAAAATGCGATATAATCCAGAAATATTCATATACAATACGAATAAACCTTTCCCGAGGTTTGATCGTATTGCTATGGAA
GCTATTTATCGGCGTAGAAATGTTTTGATTGAATGTAAAGCGAGTGAAGAGAAGAAGCGAGGATGTAAGCATTGTGAGAA
TGATATTCCTATTGCTGAATGCAGTCCTAAGATGTTGAAAGATTTTCATCATATTAAATTTAGGTATGCACATGATGTAT
GCAATTCCGAAACCACATGGTCTGAATGGATGACGTATAATGAATTTCTTGAATGGATAACTCCTGTGTATATGGCTAAC
CGTCGTAAGGCGAATGAATCGTTTAAGATGCGTGTGGATGAAATGCAAATGTTACGTATGGATGAACCATTAGAAGGTGA
TAATATTCTCAATAAGTATGTTGAAGTTAATCAGCGCTTAGTGGAGGAAATGAAGGCATTTAAGGAGCGTACACTATGGT
CAGATTTACATCGCGTAGGTGCGGAAATTAGTGCGTCAGTTAAGAAAGCTTTGCCAACCATTTCCATAACCGAAAAATTA
CCACATTGGACTGTTCAATGTGGTATTGCTAAACCTGAGATGGATCATGCTTATGAGGTTATGAGTTCGTATGCAGCTGG
AATGAATGCAGAGATTGAAGCGCATGAACAAGTTCGGCGTTCATCAGTGGAATGTCAATATGCAGAACCTCAAGCTCCAA
GAAATCCTGATGATGAAGGACCAACCATAGATGAAGAACTTATGGGCGACACTGAATTTACATCACAGGCTCTAGAACGT
CTTGTGGATGAAGGTTATATAACTGGAAAACAGAAGAAATATATAGCTACGTGGTGTAGTAAGCGTCGTGAACATATTGC
TGACTTTGATCTTGTGTGGACTGATAATTTGCGTGTGTTAAGTGCGTATGTGCATGAACGTTCGTCTTCAACTCGGCTTT
CTACGGATGACGTCAAGTTATATAAAACAATTAGCATGTTGCATCAAAAATATGACACCACAGAGTGTGCTAAATGTCAA
CATTGGTATGCTCCGTTGACTGATATCTATGTTGATGACAAGAAATTGTTTTGGTGTCAGAAAGAGAAAAAGACACTTAT
TGATGTCCGAAAATTGTCGAAAGAAGATGTGACTGTTCAATCAAAATTGATTAATTTATCTGTTCCTTGTGGTGAAGTGT
GTATGTTGCATTCAAAATATTTCAATTATCTTTTCCATAAAGCATGGTTGTTTGAGAACCCAACTTGGCGCCTAATATAT
AATGGTACCAAGAAGGGTATGCCTGAGTACTTTATGAATTGTGTGGATGAGATTTCATTAGATTCCAAATTTGGTAAAGT
GAAAGTGTGGTTGCAAGCGATCATTGATAAGTATTTAACTCGTCCCGTGAAAATGATTCGTGATTTTCTTTTCAAGTGGT
GGCCGCAAGTTGCGTATGTGTTGAGCTTGCTAGGTATAATTGGTATAACTGCGTATGAAATGAGAAATCCGAAACCAACT
TCTGAGGAATTAGTTGATCATTATGTAAATAGGCACTGTAGTTCTGATTTTTGGTCACCAGGAATGGCAACACCTCAAGG
ATTGAAATATAGTGAAGCAGTAACAGCAAGGGCACCTCGAATTCATAGATTGCCAGTTACTACTAAGCCTCAGGGATCAA
CACAACAAGTAGACGCTGCTGTGAATAAAATTTTACAGAACATGGTTTACATTGGTGTTGTTTTTCCAAAAGTGCCTGGT
AGTAAGTGGCGAGACATTAATTTTAGGTGTCTTATGCTTCATAATAGGCAATGTTTAATGTTGAGGCATTATATTGAGTC
AACTGCCGCCTTTCCTGAGGGAACTAAGTACTATTTTAAGTATATTCATAATCAAGAGACTAGAATGTCTGGGGATATTT
CTGGTATTGAAATTGATTTGTTGAATTTACCTAGATTGTATTATGGTGGTCTCGCGGGAGAGGAGTCATTTGATAGCAAT
ATCGTGCTTGTGACTATGCCTAATCGTATTCCTGAGTGTAAGAGCATTATTAAGTTTATAGCGTCACATAATGAACACAT
ACGTGCTCAGAATGATGGAGTGTTAGTAACTGGCGACCATACTCAGCTATTGGCTTTCGAGAATAATAATAAAACTCCAA
TAAGTATCAACGCTGATGGTTTGTATGAGGTTATACTTCAAGGAGTATATACTTATCCATACCATGGCGACGGTGTTTGT
GGTTCCATATTGTTATCTCGGAATTTACAACGGCCAATTATAGGTATCCATGTTGCTGGTACTGAAGGATTGCATGGCTT
TGGAGTTGCTGAACCACTTGTACATGAAATGTTCACTGGTAAAGCAATTGAGAGTGAGAGAGAACCGTATGATCGTGTGT
ATGAACTTCCGTTGCGTGAATTAGATGAATCTGATATTGGTTTAGATACTGATTTATATCCGATTGGTAGAGTGGATGCA
AAGCTAGCTCATGCTCAAAGCCCTTCTACTGGGATTAAAAAGACGCTTATCCATGGAACATTTGACGTAAGGACTGAACC
AAATCCGATGTCATCACGTGATCCAAGAATAGCGCCGCATGATCCTTTGAAGTTAGGGTGTGAAAAGCATGGAATGCCCT
GTTCACCGTTTAATAGGAAACATTTGGAATTAGCGACAAATCATTTGAAAGAAAAACTAATTTCAGTAGTTAAACCAATA
AATGGATGCAAGATTAGAAGTTTGCAAGATGCTGTATGTGGTGTGCCTGGTTTAGATGGGTTTGATTCGATATCTTGGAA
TACTAGTGCTGGCTTTCCTTTGTCTTCATTAAAGCCACCTGGAACATCAGGTAAGCGATGGTTGTTTGACATTGAGCTAC
AAGACTCGGGATGTTATCTCTTGCGTGGAATGCGTCCCGAACTTGAGATTCAATTATCAACGACACAGTTAATGAGGAAA

AAGGGAATAAAACCTCACACTATATTCACGGATTGTTTGAAAGATACTTGTTTGCCTGTGGAAAAATGTAGAATACCTGG
TAAGACTAGAATATTTAGTATAAGTCCGGTACAATTTACTATACCGTTTAGACAGTATTACTTAGATTTTATGGCATCCT
ATCGAGCTGCACGCCTTAATGCTGAGCATGGTATCGGTATTGATGTTAACAGCTTAGAATGGACAAATTTGGCAACGAGT
TTGTCGAAGTATGGCACTCATATCGTGACGGGTGACTATAAGAATTTTGGCCCTGGATTAGATTCCGATGTTGCAGCTTC
GGCGTTTGAAATTATTATCGACTGGGTATTACATTATACTGAAGAAGATAATAAAGACGAAATGAAGCGAGTAATGTGGA
CCATGGCGCAAGAGATTCTAGCGCCTAGTCATCTATGTCGTGATTTAGTGTACCGAGTACCTTGTGGAATTCCATCAGGT
TCTCCGATAACGGACATTTTGAATACAATTTCAAATTGTCTGTTAATTAGGTTAGCTTGGCTAGGTATTACTGATTTGCC
TTTATCCGAGTTCTCTCA

>Inoculum-DWV-B; IVA partial genome DWV-A contig sequence
(Row 6 of Figure S5, code: DWV-A_contig.00003-Inoculum-B)
CGGACATTTTGAATACAATTTCAAATTGTCTGTTAATTAGGTTAGCTTGGCTAGGTATTACTGATTTGCCTTTATCCGAG
TTCTCTCAAAATGTTGTTCTTGTTTGTTATGGTGATGATCTTATCATGAATGTTAGTGATAACATGATTGACAAATTTAA
TGCTGTGACAATAGGGAAATTCTTTTCACAATATAAGATGGAATTTACGGATCAGGACAAATCAGGAAATACTGTGAAGT
GGCGGACGTTACAGACTGCTACTTTCTTGAAGCATGGGTTTTTAAAACATCCGACTAGACCTGTGTTTCTGGCTAACCTA
GACAAGGTTTCGGTAGAAGGAACGACGAATTGGACCCATGCTCGGGGATTGGGTCGTCGTGCAGCAACCATAGAGAATGC
TAAACAAGCGCTAGAGTTAGCATTCGGATGGGGTCCAGAATACTTTAACTATGTCAGAAATACTATTAAAATGGCTTTTG
ACAAGTTGGGTATTTATGAAGACCTTATCACATGGGAAGAAATGGATGTTAGATGTTATGCTAGCGCGTAGTATTTAATT
TTGAATACTCATTAGTTTTAATTTTATTTTAGGTTATTGGAATTGAGGGAAGTACCACCCCCCAAGACCTTCGTTTTAAA
TCTACTAGAAGGAGTGAACCTATATATAAGAGTCTAAAGACAGAG

Figure S4. Complete genome contig sequences of the DWV-B inoculum and DWV-B derived from a white-eyed host pupa experimentally inoculated with the DWV-B inoculum as it eclosed as an adult (individual pupal code: V4-DWV-B), as well as four partial DWV-B contigs from the DWV-A inoculated pupa D4-DWV-A, as generated by the IVA *de novo* assemblies of all DWV NGS reads.

>Inoculum-DWV-B; IVA full-genome DWV-B contig sequence
(Row 9 of Figure S5, code: DWV-B_contig.00001-Inoculum-B)

```
GCTCTTCCGATCTTGGGAGGCGATTTATGCCTTCCATAGCGAATTACGGTGCAACTAACAATTTTAGATAGTAGCCATGA
ACAAACATTATGATTACTCACTACGTATTGATCATTTTTATAATGACTTGCGTAGCATGAAGCGCATGCTTGTAGTTATA
ACTATGTTATTTTGCAAGTTGGAGATAATTGTATTGGATTATGGATGCGTGCACTAAGTGTCTACATCTATAGTCGTTTG
TGGTTCAAGTTTTTGTGTTGGTAGTACAATCTTGAAGAATGTAAGTATCGTATGAATGATATTTGAATGACAACACTGAA
GTATAAAATATATAAAATCCAAAAATATTTTTAATCTTATTCAGTGTAGTGTTTGATAGAGTAGAATGCCATGTGACCGC
TCAAAGAAGTCCATTATGGTATATCATTCGAAGTCGAATACTTGTGTATAGTTATTGTATTTTATTAGTAATATTAGTAG
TCCGTAACTATCATAATCCTATTATAGTTTGATTATATGATAGACCACTGCAGTATCGAGTAGAGTTTAGAAAGAGTAGT
GCAATAGTAAGATCACTGTCACCGACCACTCATTGTAATAGTGAGGTTTGTCGGAAACCAGTTATTGTGCAGCGACTAGC
AATCGTGAATCAATATAGTTGGTATTCTAAATATGAGACGATTCGGCGATTTTATTGCGACTGAAATTTCATATTTAGCA
TGTCAGGTCTTATTATGAATGCTCGAGTATTTATTTCTGCGGTAGAGTAGGGACCCCTCTATCTCTCAGGTACTGTATGA
GGCGAAAGTGTGAAAGTAATTTATGTCTTTATACATAAGTGACTGTATCGGGATTTCCTTTGGCAAGAATCCTTTTAATA
CAGTATAATTTATGTCACGGTACGTTACGTTCGCAGGGCACCCGTTAATGTCACATAGCCCAGACGATGACGAATGGAAA
GACATTACTTTTTATTTTAATGCTACGATTATTGCTGTTTTATTTTGCTGCTTTTATTTGCTATTATATTTTGCTATTTT
CATTATTGCTAAATATATTTCTTTGCTATTTTTTCTTTATATATTAGATTCAATTCTTTTTATTTTATATTTTCAATTTG
ATTTTGATTTTGAAGGTAAATATATATAAAAATGGCATTTAGTTGTGGAACTCTTTCTTATGCTGCTGTTGCCCAAGCTC
CCTCTGTAGCTCATGCTCCCCGTAGTTGGGAGATTGATGAAGCTAGGCGTCGACGCGTTATTAAGCGTTTGGCGTTGGAA
CAGGAACGGATTCGAAACGTTCTTGACGTCACTGTGTATGATCATACAACGTGGGAGCAAGAAGATGCGCGTGATAATGA
GTTCCTTACGGAACAATTGAATAATTTATATACGATATATTCTATAGCTGAAAGATGTACCCGCCGTCCTGTTCAAGAAC
ATGTCCCCATTTCAATCAGTAATAGATATTCCCCTTTAGAATCCCTTAAGATTGAGGTAGGAAAAGACGCGGGTGAGTTC
GTATTTAAGAAACCCAAATATACAAAGATTTGTAAGAAAGTGAAACGGGTGGCATCAAAATTTGTGCGCGAGAAAGTTGT
TAGGCCCGTTTGTAATCGATCGCCTATGTTATTATTTAAAATTAAGAAAGTAATATATGATTTACATTTGTATCGGTTAC
GGAAACAAGTTCGGCTTCTCAGACGCGAAAAACAGCGTGAATACGAGTTAGAGTGTGTTACTAGTTTGCTACAGCTATCT
AATCCTGTTTCAGCTAAACCTGAGATGGACAATCCTAATCCTGGTCCAGATGGTGAAGGTGAAGTTGAATTAGAAAAGGA
TAGTAATGTAGTATTAACTACACAACGTGATCCTAGTACCTCTATTCCTGCTCCAACTAGTGTGAAGTGGAGTAGATGGA
CTAGTAATGATGTTGTGGATGATTATGCCACTATAACTTCGCGCTGGTATCAAATTGCCGAATTTGTATGGTCAAAGGAT
GATCCATTTGATAAGGAATTGGCGCGCTTAATTTTACCTCGAGCTTTGTTATCTAGTATTGAGGCTAATTCTGACGCTAT
TTGTGATGTACCTAATACTATTCCGTTTAAGGTACATGCATATTGGCGTGGAGATATGGAAGTTCGAGTGCAGATTAACT
CGAATAAATTCCAGGTTGGTCAATTACAGGCAACTTGGTACTATTCGGATCATGAAAATTTGAATATTCAGACGAAGCGA
AGTGTGTATGGTTTTTCGCATATGGATCATGCTCTGATTAGCGCATCAGCGAGTAATGAAGCAAGATTAGTGATACCTTT
TAAACACGTATATCCATTCTTACCAACGCGTGTCGTTCCTGATTGGACAACTGGTATTCTTGATATGGGTACCTTAAATA
TTCGTGTAATTGCTCCACTACGTATGAGTGCGACGGGACCAACCACTTGTAATGTTGTAGTATTTATTAAGTTAAATAAT
AGTGAATTCACTGGTACTTCTTCTGGTAAGTTTTACGCAAATCAAATTAGGGCAAAACCTGAAATGGACCGTGTGTTAAA
TTTGGCAGAAGGATTACTAAATAATACTGTAGGTGGTTGTAATATGGATAATCCGTCATATCAGCAATCTCCGCGTCATT
TTGTTCCTACTGGTATGCATAGTTTAGCTTTAGGCACTAATTTAGTAGAGCCTTTGCATGCATTACGATTAGATGCATCA
GGTACAACACAACATCCAGTTGGGTGTGCGCCTGATGAAGATATGACTGTATCTTCCATTGCATCACGATATGGTTTAAT
TCGCCAAGTGCAATGGAAGAAAGACCATGCGAAAGGATCATTATTATTACAACTTGATGCTGATCCTTTCGTTGAACAGA
AAATTGAGGGAACCAATCCAATTTCTTTGTATTGGTTTGCTCCGGTTGGAGTCGTATCTAGTATGTTTATGCAATGGAGA
GGTTCTTTAGAATATAGATTTGATATTATAGCTTCCCAATTTCATACGGGTAGGTTAATTGTAGGTTATGTTCCTGGACT
GACTGCTTCTTTACAACGTCAAATGGACTATATGAAATTGAAGTCATCTAGTTATGTGGTGTTTGATTTACAGGAAAGTA
ATAGTTTTACGTTTGAAGTGCCCTATGTGTCATACAGACCGTGGTGGGTGCGTAAGTATGGTGGTAATTATCTACCATCC
TCTACTGATGCGCCTAGCACACTGTTTATGTATGTACAAGTACCATTGATACCTATGGAAGCTGTTTCTGATACTATAGA
TATCAATGTGTATGTGCGTGGTGGCAGTTCGTTTGAGGTTTGTGTTCCAGTCCAACCTAGTTTAGGTTTGAATTGGAATA
CAGATTTCATATTACGTAATGATGAGGAGTACCGCGCAAAGAATGGATATGCACCATATTATGCTGGTGTGTGGCATAGC
TTCAATAATAGTAATTCGCTTGTTTTTAGATGGGGTTCGGCTTCAGATCAAATTGCTCAATGGCCAACAATAACAGTGCC
TCGAGGAGAGTTGGCATTCCTGCGTATCCGCGATGCTAAGCAAGCTGCTGTAGGAACGCAACCTTGGCGTACTATGGTCG
TTTGGCCTTCAGGTCATGGATATAACATTGGAATACCAACTTATAATGCTGAACGAGCAAGACAACTTGCTCAGCATTTG
TATGGTGGTGGGTCTTTGACAGATGAAAAGGCTAAGCAATTATTTGTGCCTGCTAACCAGCAAGGACCCGGCAAAGTAAG
TAATGGTAACCCCGTCTGGGAAGTAATGCGCGCGCCTCTTGCAACTCAGCAAGCGCATATACAAGATTTTGAATTTGTTG
AAGCTGTTCCAGAAGGCGAAGAATCACGCAACACTACGGTGCTAGACACGACAACAACGTTACAGTCTAGCGGATTTGGT
CGCGCTTTCTTCGGTGAGGCATTTAACGATCTTAAGACGTTAATGCGCCGATACCAATTATATGGTCAATTATTGTTATC
```

CGTTACTACGGATAAGGATATTGATCATTGTATGTTTACCTTCCCTTGTTTACCTCAAGGGTTAGCGTTAGATATAGGTT
CGGCTGGATCTCCTCATGAAATATTTAATCGCTGCCGTGATGGTATCATTCCATTGATAGCGTCAGGGTATCGGTTTTAT
CGAGGCGATTTACGGTTCAAAATTGTTTTCCCAAGTAACGTTAATAGCAATATTTGGGTACAACATCGACCAGATCGTAG
ACTGAAAGGATGGTCTGAAGCGAAAATAGTAAACTGTGATGCTGTATCTACTGGACAAGGTGTTTATAATCATGGATATG
CTAGTCATATTCAGATTACGCGTGTAAATAATGTTATAGAATTGGAAGTTCCGTTTTATAACGCTACGTGCTATAATTAT
TTGCAAGCGTTTAACCCATCTAGTGCAGCGTCGAGTTATGCCGTTTCGCTCGGAGAGATTTCGGTTGGTTTTCAAGCTAC
TAGTGATGATATTGCAGCCATAGTTAATAAACCTGTAACTATATATTACAGTATTGGCGATGGTATGCAGTTTTCGCAGT
GGGTTGGTTATCAACCAATGATGATTCTAGATCAATTGCCAGCACCAGTAGTTAGGGCTGTGCCTGAGGGCCCTATAGCT
AAGATAAAGAACTTTTTCCACCAAACGGCAGATGAAGTTCGAGAAGCTCAGGCCGCAAAGATGCGTGAAGATATGGGTAT
AGTAGTCCAAGACGTCATAGGAGAGTTAAGTCAGGCTATACCCGATCTTCAACAACCGGAAGTTCAAGCGAATGTTTTTT
CTCTGGTGTCACAGTTAGTGCATGCTATCATCGGTACTAGTCTTAAGACAGTTGCTTGGGCGATTGTTTCGATTTTTGTA
ACTTTAGGTTTGATTGGACGTGAAATGATGCATTCAGTCATAACTGTAGTTAAGCGGTTATTAGAAAAATATCACTTGGC
GACGCAACCCCAGGAATCCGCCAATTCAGGTACGGTTATTTCCGCTATTCCAGAAGCACCCAATGCTGAAGCAGAGGAGG
CCAGTGCCTGGGTATCCATTATTTATAATGGTGTGTGTAATATGTTGAATGTAGCCGCTCAAAAACCGAAACAATTTAAA
GATTGGGTAAAATTAGCTACCGTAGATTTTAGTAATAATTGTAGAGGTAGTAATCAGGTATTTGTGTTTTTCAAGAATAC
GTTTGAAGTGTTGAAGAAGATGTGGGGTTATGTGTTTTGTCAGAGTAATCCTGCAGCGCGACTCTTGAAAGCAGTGAATG
ATGAACCTGAGATTTTAAAGGCGTGGGTTAAAGAATGTCTGTATTTAGATGATCCTAAATTTAGAATGCGACGTGCGCAT
GATCAAGAGTATATTGAGAGAGTGTTTGCGGCCCATTCGTATGGACAAATTTTATTGCATGACTTAACGGCTGAAATGAA
TCAATCGCGTAATTTAAGTGTGTTTACGAGAGTGTACGATCAAATATCTAAATTGAAGACGGATCTCATGGAAATGGGAT
CAAACCCATATATCAGGCGTGAATGCTTTACGATTTGTATGTGTGGTGCATCTGGAATTGGTAAGTCTTATTTAACTGAT
TCTTTATGCAGCGAGCTCTTACGTGCGAGTCGTACTCCAGTGACAACGGGCATTAAGTGTGTCGTGAACCCTTTGTCTGA
TTATTGGGATCAGTGTGATTTTCAGCCCGTTTTATGTGTTGATGACATGTGGAGTGTTGAAACGTCTACTACGCTCGATA
AACAGTTAAATATGCTATTTCAGGTTCATTCACCAATTGTACTTTCACCTCCTAAAGCTGATTTAGAAGGTAAGAAAATG
CGTTATAATCCTGAAATATTCATATATAATACGAATAAACCTTTTCCGAGGTTTGATCGTATAGCTATGGAAGCTATTTA
TCGACGTAGAAACGTTTTAATTGAATGTAAGGCTAATGAAGAGAAGAAGCGTGGATGTAAACATTGTGAGAATAATATAC
CCATTGCTGAATGTAGTCCAAAAATTTTGAAAGATTTTCATCACATTAAATTTCGTTATGCTCATGATGTGTGTAATTCT
GAAACTACGTGGTCTGAGTGGATGTCGTATAATGAATTTTTGGAATGGATTACTCCCGTATATATGGCTAATCGACGTAA
AGCAAATGAATCGTTTAAGATGCGTGTTGATGAAATGCAAATGTTGCGTATGGATGAGCCCTTGGAAGGCGATAATATTT
TAAATAAGTATGTTGAAGTTAATCAGCGCTTAGTTGAGGAAATGAAAGCTTTTAAAGAGCGAACCCTCTGGGCTGATTTA
CAACGTGTTGGCTCAGAGATTAGTACTTCAGTTAAGAAAGCATTACCAACTATTTCCATTACTGAGAAGCTACCACATTG
GACTATCCAATGTGGCATAGCTAAGCCTGAAATGGATCATGCTTATGAAGTTATGAGTTCATATGCAGCAGGAATGAACG
CAGAAATTGAAGCGCATGAACAAGTTCGTCGTTCTTCTTTGGAATGTCAGTGTATTGAGCCCTCAACTTCAAGACCTCTG
GATGAAGAGGGTCCTACTATCGACGAGGAATTACTTGGCGAAGTAGAATTTACTTCTTCAGCTTTGGAGCGTTTGGTTGA
TGAGGGGTATATTACTGGTAAACAAAAGAAGTACATGGCAACTTGGTGTACGAAGCGAAGAGAGCATGTATCCGATTTTG
ATTTAGTATGGACGGATAATTTGCGTGTTTTGAGTGCGTATGTCCACGAGCGTTCTACATCTACGCGTTTATCTACCGAT
GATGTTAAATTATTTAAGACGATTAGTATGTTACATCAGAGGTATGACACCACTGATTGTGCAAAATGCCAACATTGGTA
TGCACCATTAACAGCTATTTATGTTGATGATAGAAAGCTATTTTGGTGCCAGAAGGAGACTAAGACTTTGATAGATGTTC
GTAAATTGTCGAAAGAGGACGTTACAGTCCAATCGAAATTAATTAACTTATCGGTTCCGTGCGGTGATGTATGTATGTTA
CATTCTAAGTACTTTAATTATTTATTCCATAAAGCGTGGTTGTTTGAAAATCCAACATGGCGTTTAATATATAATGGTAC
TAAGAAAGGTATGCCTGAGTATTTCATGAATTGCGTGGATGAAATTTCATTAGATTCAAAATTTTGTAAAGTAAAGGTTT
GGCTTCAAGCAATTATTGATAAATATTTGACTCGTCCAGTGAAAATGATTCGTGACTTTCTATTTAAATGGTGGCCGCAA
GTAGCATACGTGTTAAGTTTGTTAGGTATAATTGGTATAACTGCGTATGAGATGCGTAATCCTAAATCAACAGCAGAAGA
CTTGGCTGAGCACTATGTTAATAGGCATTGTAGTTCAGATTTTTGGTCACCAGGTATGGCGACTCCTCAGGGATTAAAAT
ATAGTGAAGCGATAACAGCTAAAGCGCCTAGAATCCATAGATTGCCCGTTACTACTAGACCTCAGGGATCAACGCAACAA
GTTGATGCCGCTGTGAATAAGATTTTGCAGAATATGGTGTATATCGGTGTTGTGTTTCCGAAAGTGCCTGGTAGTAAGTG
GCGAGATATTAATTTTAGATGTCTTATGCTTCATAATCGGCAATGTTTGATGTTGCGGCATTACATTGAGTCGACGGCTG
CTTTTCCGGAGGGTACCAAATACTATTTTAAGTATATTCATAATCAAGAAACTCGAATGTCAGGTGATATATCTGGTATT
GAGATTGATTTATTGAGTTTACCTAGATTGTATTATGGTGGCTTAGCTGGGGAAGAGTCGTTCGATAGCAATATAGTGTT
AGTAACTATGCCGAATAGAATTCCTGAGTGTAAGAGTATTGTGAAGTTTATAGCTTCACATGCTGAACATGCTCGTGCTC
AAAATGATGGTGTGTTAGTTACTGGTGAACATACCCAGTTATTGGCGTTCGAGAATAATAATAAAACACCTATAAGTATT
AATGCTGATGGTTTGTATGAGGTTATACTTCAAGGAGTATACACTTATCCATACCATGGTGATGGTGTTTGTGGGTCTAT
ATTATTGTCTCGTAATTTACAACGACCGATTATAGGGATCCATGTAGCTGGTACTGAAGGATTACATGGCTTTGGTGTTG
CTGAACCTCTTGTTCATGAGATGTTCACTGGGAAAGCAATAGAGAGTGAAAGGGAACCGTATGATCGTGTGTATGAATTA
CCTTTGCGTGAATTAGATGAATCTGATATAGGTTTAGATACTGATTTATATCCTATAGGAAGAGTTGATGCGAAATTAGC
CCATGCCCAAAGTCCTTCAACAGGAATTAAAAAGACGCTTATTCATGGTACTTTTGATGTTCGGACTGAACCGAATCCGA
TGTCATCACGAGACCCAAGAATAGCGCCACATGATCCGTTGAAGTTAGGGTGTGAGAAACATGGTATGCCATGTTCTCCA
TTTAATCGAAAACATTTGGAATTAGCAACAACTCATTTAAAGGAGAAGTTAATTTCCGTAGTTAAACCTATAAACGGATG
CAAGATTAGAAGTTTGCAAGATGCTGTGTGTGGTGTACCAGGTTTGGATGGCTTTGATTCAATATCCTGGAATACTAGTG
CTGGTTTTCCTTTATCTTCATTAAAACCACCAGGCTCTTCTGGTAAGCGATGGTTGTTTGATATTGAATTACAAGATTCA
GGATGTTATCTTTTGAGAGGGATGAGACCTGAACTTGAGATACAGTTGACAACAACTCAGTTAATGAGGAAGAAGGGAAT
AAAGCCTCACACTATATTCACGGATTGTTTAAAAGATACATGTTTGCCTGTGGAAAAATGCAGAATACCTGGTAAGACTA
GAATATTTAGTATAAGCCCCGTCCAATTTACGATTCCATTCCGACAATACTATCTCGATTTTATGGCGTCGTACCGTGCC
GCTAGACTTAATGCTGAGCATGGAATAGGTATAGACGTGAACAGCTTGGAGTGGACAAACTTGGCAACAAGTCTGTCGAA

GTATGGCACGCATATTGTGACAGGAGATTACAAGAATTTTGGTCCTGGGTTAGATTCTGATGTTGCCGCTTCAGCTTTCG
AAATTATCATTGATTGGGTGTTAAATTACACTGAAGAAGATGATAAAGACGAAATGAAGCGTGTAATGTGGACTATGGCT
CAGGAAATTTTAGCTCCTAGTCACTTATGTCGTGATTTAGTATATCGCGTACCATGCGGTATTCCTTCTGGATCACCAAT
TACGGACATTTTGAATACTATTTCGAATTGTTTGTTAATTCGATTGGCTTGGCAAGGTATTACCGATTTGCCTTTATCCG
AATTTTCTAGACATGTCGTGCTAGTTTGTTACGGTGATGATCTCATCATGAATGTAAGTGATGAAATGATAGACAAATTC
AACGCTGTAACAATTGGTGATTTCTTTTCGCGATATAAGATGGAATTTACGGATCAGGATAAATCTGGAAATACAGTGCG
GTGGCGAACTTTACAAACTGCCACCTTTTTGAAGCATGGGTTCTTGAAACATCCAACAAGACCCGTGTTTCTAGCCAATC
TGGATAAGGTTTCTATAGAAGGAACAACCAATTGGACACATGCTCGAGGATTGGGTCGTCGAGTAGCAACCATTGAGAAT
GCTAAACAAGCGCTAGAGTTGGCATTCGGATGGGGTCCCGAATACTTTAATCATGTTCGGAATACCATTAAAATGGCATT
CGACAAGTTAGGTATTTATGAGGACCTCATCACATGGGAAGAAATGGATGTTAGATGTTATGCTAGCGCGTAATTTTAAG
ATTTTAATACTCATTAAAATTAATTTATATTTAGGTTATTGGAATTGAGGGAAGTACCACCCCCCAAGACCTTCGTTTTA
AATCTACTAAGAGGAGTGAACTTGCATATAAGAGTCTAAAAGCAGAGTGGATTAGACCACCACTTTTAGCTTATATGTGA
GGAAGGTTGAGTTGCCTCTAAAGACTCAGCTCCGTAGTAGAGTAGTTTTAGTTACGATTAAAGTGGTACTCTAGGTTAGG
TGTTACTCGCGCATTGTCGCATAACGGCAATGCGTCCTAATTTTAGTATAGTTTAACCATAATAGTAAAAAAAAAAAAAA
AAAAAAAAAAAAAA

> Code of pupa: V4-DWV-B; IVA full genome DWV-B contig sequence
(Row 10 of Figure S5, code: DWV-B_contig.00001-V4-B)

CTATGGGAGGCGATTTATGCCTTCCATAGCGAATTACGGTGCAACTAACAATTTTAGATAGTAGCCATGAACAAACATTA
TGATTACTCACTACGTATTGATCATTTTTATAATGACTTGCGTAGCATGAAGCGCATGCTTGTAGTTATAACTATGTTAT
TTTGCAAGTTGGAGATAATTGTATTGGATTATGGATGCGTGCACTAAGTGTCTACATCTATAGTCGTTTGTGGTTCAAGT
TTTTGTGTTGGTAGTACAATCTTGAAGAATGTAAGTATCGTATGAATGATATTTGAATGACAACACTGAAGTATAAAATA
TATAAAATCCAAAAATATTTTTAATCTTATTCAGTGTAGTGTTTGATAGAGTAGAATGCCATGTGACCGCTCAAAGAAGT
CCATTATGGTATATCATTCGAAGTCGAATACTTGTGTATAGTTATTGTATTTTATTAGTAATATTAGTAGTCCGTAACTA
TCATAATCCTATTATAGTTTGATTATATGATAGACCACTGCAGTATCGAGTAGAGTTTAGAAAGAGTAGTGCAATAGTAA
GATCACTGTCACCGACCACTCATTGTAATAGTGAGGTTTGTCGGAAACCAGTTATTGTGCAGCGACTAGCAATCGTGAAT
CAATATAGTTGGTATTCTAAATATGAGACGATTCGGCGATTTTATTGCGACTGAAATTTCATATTTAGCATGTCAGGTCT
TATTATGAATGCTCGAGTATTTATTTCTGCGGTAGAGTAGGGACCCCTCTATCTCTCAGGTACTGTATGAGGCGAAAGTG
TGAAAGTAATTTATGTCTTTATACATAAGTGACTGTATCGGGATTTCCTTTGGCAAGAATCCTTTTAATACAGTATAATT
TATGTCACGGTACGTTACGTTCGCAGGGCACCCGTTAATGTCACATAGCCCAGACGATGACGAATGGAAAGACATTACTT
TTTATTTTAATGCTACGATTATTGCTGTTTTATTTTGCTGCTTTTATTTGCTATTATATTTTGCTATTTTCATTATTGCT
AAATATATTTCTTTGCTATTTTTTCTTTATATATTAGATTCAATTCTTTTTATTTTATATTTTCAATTTGATTTTGATTT
TGAAGGTAAATATATATAAAAATGGCATTTAGTTGTGGAACTCTTTCTTATGCTGCTGTTGCCCAAGCTCCCTCTGTAGC
TCATGCTCCCCGTAGTTGGGAGATTGATGAAGCTAGGCGTCGACGCGTTATTAAGCGTTTGGCGTTGGAACAGGAACGGA
TTCGAAACGTTCTTGACGTCACTGTGTATGATCATACAACGTGGGAGCAAGAAGATGCGCGTGATAATGAGTTCCTTACG
GAACAATTGAATAATTTATATACGATATATTCTATAGCTGAAAGATGTACCCGCCGTCCTGTTCAAGAACATGTCCCCAT
TTCAATCAGTAATAGATATTCCCCTTTAGAATCCCTTAAGATTGAGGTAGGAAAAGACGCGGGTGAGTTCGTATTTAAGA
AACCCAAATATACAAAGATTTGTAAGAAAGTGAAACGGGTGGCATCAAAATTTGTGCGCGAGAAAGTTGTTAGGCCCGTT
TGTAATCGATCGCCTATGTTATTATTTAAAATTAAGAAAGTAATATATGATTTACATTTGTATCGGTTACGGAAACAAGT
TCGGCTTCTCAGACGCGAAAAACAGCGTGAATACGAGTTAGAGTGTGTTACTAGTTTGCTACAGCTATCTAATCCTGTTT
CAGCTAAACCTGAGATGGACAATCCTAATCCTGGTCCAGATGGTGAAGGTGAAGTTGAATTAGAAAAGGATAGTAATGTA
GTATTAACTACACAACGTGATCCTAGTACCTCTATTCCTGCTCCAACTAGTGTGAAGTGGAGTAGATGGACTAGTAATGA
TGTTGTGGATGATTATGCCACTATAACTTCGCGCTGGTATCAAATTGCCGAATTTGTATGGTCAAAGGATGATCCATTTG
ATAAGGAATTGGCGCGCTTAATTTTACCTCGAGCTTTGTTATCTAGTATTGAGGCTAATTCTGACGCTATTTGTGATGTA
CCTAATACTATTCCGTTTAAGGTACATGCATATTGGCGTGGAGATATGGAAGTTCGAGTGCAGATTAACTCGAATAAATT
CCAGGTTGGTCAATTACAGGCAACTTGGTACTATTCGGATCATGAAAATTTGAATATTCAGACGAAGCGAAGTGTGTATG
GTTTTTCGCATATGGATCATGCTCTGATTAGCGCATCAGCGAGTAATGAAGCAAGATTAGTGATACCTTTTAAACACGTA
TATCCATTCTTACCAACGCGTGTCGTTCCTGATTGGACAACTGGTATTCTTGATATGGGTACCTTAAATATTCGTGTAAT
TGCTCCACTACGTATGAGTGCGACGGGACCAACCACTTGTAATGTTGTAGTATTTATTAAGTTAAATAATAGTGAATTCA
CTGGTACTTCTTCTGGTAAGTTTTACGCAAATCAAATTAGGGCAAAACCTGAAATGGACCGTGTGTTAAATTTGGCAGAA
GGATTACTAAATAATACTGTAGGTGGTTGTAATATGGATAATCCGTCATATCAGCAATCTCCGCGTCATTTTGTTCCTAC
TGGTATGCATAGTTTAGCTTTAGGCACTAATTTAGTAGAGCCTTTGCATGCATTACGATTAGATGCATCAGGTACAACAC
AACATCCAGTTGGGTGTGCGCCTGATGAAGATATGACTGTATCTTCCATTGCATCACGATATGGTTTAATTCGCCAAGTG
CAATGGAAGAAAGACCATGCGAAAGGATCATTATTATTACAACTTGATGCTGATCCTTTCGTTGAACAGAAAATTGAGGG
AACCAATCCAATTTCTTTGTATTGGTTTGCTCCGGTTGGAGTCGTATCTAGTATGTTTATGCAATGGAGAGGTTCTTTAG
AATATAGATTTGATATTATAGCTTCCCAATTTCATACGGGTAGGTTAATTGTAGGTTATGTTCCTGGACTGACTGCTTCT
TTACAACGTCAAATGGACTATATGAAATTGAAGTCATCTAGTTATGTGGTGTTTGATTTACAGGAAAGTAATAGTTTTAC
GTTTGAAGTGCCCTATGTGTCATACAGACCGTGGTGGGTGCGTAAGTATGGTGGTAATTATCTACCATCCTCTACTGATG
CGCCTAGCACACTGTTTATGTATGTACAAGTACCATTGATACCTATGGAAGCTGTTTCTGATACTATAGATATCAATGTG
TATGTGCGTGGTGGCAGTTCGTTTGAGGTTTGTGTTCCAGTCCAACCTAGTTTAGGTTTGAATTGGAATACAGATTTCAT
ATTACGTAATGATGAGGAGTACCGCGCAAAGAATGGATATGCACCATATTATGCTGGTGTGTGGCATAGCTTCAATAATA
GTAATTCGCTTGTTTTTAGATGGGGTTCGGCTTCAGATCAAATTGCTCAATGGCCAACAATAACAGTGCCTCGAGGAGAG

TTGGCATTCCTGCGTATCCGCGATGCTAAGCAAGCTGCTGTAGGAACGCAACCTTGGCGTACTATGGTCGTTTGGCCTTC
AGGTCATGGATATAACATTGGAATACCAACTTATAATGCTGAACGAGCAAGACAACTTGCTCAGCATTTGTATGGTGGTG
GGTCTTTGACAGATGAAAAGGCTAAGCAATTATTTGTGCCTGCTAACCAGCAAGGACCCGGCAAAGTAAGTAATGGTAAC
CCCGTCTGGGAAGTAATGCGCGCGCCTCTTGCAACTCAGCAAGCGCATATACAAGATTTTGAATTTGTTGAAGCTGTTCC
AGAAGGCGAAGAATCACGCAACACTACGGTGCTAGACACGACAACAACGTTACAGTCTAGCGGATTTGGTCGCGCTTTCT
TCGGTGAGGCATTTAACGATCTTAAGACGTTAATGCGCCGATACCAATTATATGGTCAATTATTGTTATCCGTTACTACG
GATAAGGATATTGATCATTGTATGTTTACCTTCCCTTGTTTACCTCAAGGGTTAGCGTTAGATATAGGTTCGGCTGGATC
TCCTCATGAAATATTTAATCGCTGCCGTGATGGTATCATTCCATTGATAGCGTCAGGGTATCGGTTTTATCGAGGCGATT
TACGGTTCAAAATTGTTTTCCCAAGTAACGTTAATAGCAATATTTGGGTACAACATCGACCAGATCGTAGACTGAAAGGA
TGGTCTGAAGCGAAAATAGTAAACTGTGATGCTGTATCTACTGGACAAGGTGTTTATAATCATGGATATGCTAGTCATAT
TCAGATTACGCGTGTAAATAATGTTATAGAATTGGAAGTTCCGTTTTATAACGCTACGTGCTATAATTATTTGCAAGCGT
TTAACCCATCTAGTGCAGCGTCGAGTTATGCCGTTTCGCTCGGAGAGATTTCGGTTGGTTTTCAAGCTACTAGTGATGAT
ATTGCAGCCATAGTTAATAAACCTGTAACTATATATTACAGTATTGGCGATGGTATGCAGTTTTCGCAGTGGGTTGGTTA
TCAACCAATGATGATTCTAGATCAATTGCCAGCACCAGTAGTTAGGGCTGTGCCTGAGGGCCCTATAGCTAAGATAAAGA
ACTTTTTCCACCAAACGGCAGATGAAGTTCGAGAAGCTCAGGCCGCAAAGATGCGTGAAGATATGGGTATAGTAGTCCAA
GACGTCATAGGAGAGTTAAGTCAGGCTATACCCGATCTTCAACAACCGGAAGTTCAAGCGAATGTTTTTTCTCTGGTGTC
ACAGTTAGTGCATGCTATCATCGGTACTAGTCTTAAGACAGTTGCTTGGGCGATTGTTTCGATTTTTGTAACTTTAGGTT
TGATTGGACGTGAAATGATGCATTCAGTCATAACTGTAGTTAAGCGGTTATTAGAAAAATATCACTTGGCGACGCAACCC
CAGGAATCCGCCAATTCAGGTACGGTTATTTCCGCTATTCCAGAAGCACCCAATGCTGAAGCAGAGGAGGCCAGTGCCTG
GGTATCCATTATTTATAATGGTGTGTGTAATATGTTGAATGTAGCCGCTCAAAAACCGAAACAATTTAAAGATTGGGTAA
AATTAGCTACCGTAGATTTTAGTAATAATTGTAGAGGTAGTAATCAGGTATTTGTGTTTTTCAAGAATACGTTTGAAGTG
TTGAAGAAGATGTGGGGTTATGTGTTTTGTCAGAGTAATCCTGCAGCGCGACTCTTGAAAGCAGTGAATGATGAACCTGA
GATTTTAAAGGCGTGGGTTAAAGAATGTCTGTATTTAGATGATCCTAAATTTAGAATGCGACGTGCGCATGATCAAGAGT
ATATTGAGAGAGTGTTTGCGGCCCATTCGTATGGACAAATTTTATTGCATGACTTAACGGCTGAAATGAATCAATCGCGT
AATTTAAGTGTGTTTACGAGAGTGTACGATCAAATATCTAAATTGAAGACGGATCTCATGGAAATGGGATCAAACCCATA
TATCAGGCGTGAATGCTTTACGATTTGTATGTGTGGTGCATCTGGAATTGGTAAGTCTTATTTAACTGATTCTTTATGCA
GCGAGCTCTTACGTGCGAGTCGTACTCCAGTGACAACGGGCATTAAGTGTGTCGTGAACCCTTTGTCTGATTATTGGGAT
CAGTGTGATTTTCAGCCCGTTTTATGTGTTGATGACATGTGGAGTGTTGAAACGTCTACTACGCTCGATAAACAGTTAAA
TATGCTATTTCAGGTTCATTCACCAATTGTACTTTCACCTCCTAAAGCTGATTTAGAAGGTAAGAAAATGCGTTATAATC
CTGAAATATTCATATATAATACGAATAAACCTTTTCCGAGGTTTGATCGTATAGCTATGGAAGCTATTTATCGACGTAGA
AACGTTTTAATTGAATGTAAGGCTAATGAAGAGAAGAAGCGTGGATGTAAACATTGTGAGAATAATATACCCATTGCTGA
ATGTAGTCCAAAAATTTTGAAAGATTTTCATCACATTAAATTTCGTTATGCTCATGATGTGTGTAATTCTGAAACTACGT
GGTCTGAGTGGATGTCGTATAATGAATTTTTGGAATGGATTACTCCCGTATATATGGCTAATCGACGTAAAGCAAATGAA
TCGTTTAAGATGCGTGTTGATGAAATGCAAATGTTGCGTATGGATGAGCCCTTGGAAGGCGATAATATTTTAAATAAGTA
TGTTGAAGTTAATCAGCGCTTAGTTGAGGAAATGAAAGCTTTTAAAGAGCGAACCCTCTGGGCTGATTTACAACGTGTTG
GCTCAGAGATTAGTACTTCAGTTAAGAAAGCATTACCAACTATTTCCATTACTGAGAAGCTACCACATTGGACTATCCAA
TGTGGCATAGCTAAGCCTGAAATGGATCATGCTTATGAAGTTATGAGTTCATATGCAGCAGGAATGAACGCAGAAATTGA
AGCGCATGAACAAGTTCGTCGTTCTTCTTTGGAATGTCAGTGTATTGAGCCCTCAACTTCAAGACCTCTGGATGAAGAGG
GTCCTACTATCGACGAGGAATTACTTGGCGAAGTAGAATTTACTTCTTCAGCTTTGGAGCGTTTGGTTGATGAGGGGTAT
ATTACTGGTAAACAAAAGAAGTACATGGCAACTTGGTGTACGAAGCGAAGAGAGCATGTATCCGATTTTGATTTAGTATG
GACGGATAATTTGCGTGTTTTGAGTGCGTATGTCCACGAGCGTTCTACATCTACGCGTTTATCTACCGATGATGTTAAAT
TATTTAAGACGATTAGTATGTTACATCAGAGGTATGACACCACTGATTGTGCAAAATGCCAACATTGGTATGCACCATTA
ACAGCTATTTATGTTGATGATAGAAAGCTATTTTGGTGCCAGAAGGAGACTAAGACTTTGATAGATGTTCGTAAATTGTC
GAAAGAGGACGTTACAGTCCAATCGAAATTAATTAACTTATCGGTTCCGTGCGGTGATGTATGTATGTTACATTCTAAGT
ACTTTAATTATTTATTCCATAAAGCGTGGTTGTTTGAAAATCCAACATGGCGTTTAATATATAATGGTACTAAGAAAGGT
ATGCCTGAGTATTTCATGAATTGCGTGGATGAAATTTCATTAGATTCAAAATTTTGTAAAGTAAAGGTTTGGCTTCAAGC
AATTATTGATAAATATTTGACTCGTCCAGTGAAAATGATTCGTGACTTTCTATTTAAATGGTGGCCGCAAGTAGCATACG
TGTTAAGTTTGTTAGGTATAATTGGTATAACTGCGTATGAGATGCGTAATCCTAAATCAACAGCAGAAGACTTGGCTGAG
CACTATGTTAATAGGCATTGTAGTTCAGATTTTTGGTCACCAGGTATGGCGACTCCTCAGGGATTAAAATATAGTGAAGC
GATAACAGCTAAAGCGCCTAGAATCCATAGATTGCCCGTTACTACTAGACCTCAGGGATCAACGCAACAAGTTGATGCCG
CTGTGAATAAGATTTTGCAGAATATGGTGTATATCGGTGTTGTGTTTCCGAAAGTGCCTGGTAGTAAGTGGCGAGATATT
AATTTTAGATGTCTTATGCTTCATAATCGGCAATGTTTGATGTTGCGGCATTACATTGAGTCGACGGCTGCTTTTCCGGA
GGGTACCAAATACTATTTTAAGTATATTCATAATCAAGAAACTCGAATGTCAGGTGATATATCTGGTATTGAGATTGATT
TATTGAGTTTACCTAGATTGTATTATGGTGGCTTAGCTGGGGAAGAGTCGTTCGATAGCAATATAGTGTTAGTAACTATG
CCGAATAGAATTCCTGAGTGTAAGAGTATTGTGAAGTTTATAGCTTCACATGCTGAACATGCTCGTGCTCAAAATGATGG
TGTGTTAGTTACTGGTGAACATACCCAGTTATTGGCGTTCGAGAATAATAATAAAACACCTATAAGTATTAATGCTGATG
GTTTGTATGAGGTTATACTTCAAGGAGTATACACTTATCCATACCATGGTGATGGTGTTTGTGGGTCTATATTATTGTCT
CGTAATTTACAACGACCGATTATAGGGATCCATGTAGCTGGTACTGAAGGATTACATGGCTTTGGTGTTGCTGAACCTCT
TGTTCATGAGATGTTCACTGGGAAAGCAATAGAGAGTGAAAGGGAACCGTATGATCGTGTGTATGAATTACCTTTGCGTG
AATTAGATGAATCTGATATAGGTTTAGATACTGATTTATATCCTATAGGAAGAGTTGATGCGAAATTAGCCCATGCCCAA
AGTCCTTCAACAGGAATTAAAAAGACGCTTATTCATGGTACTTTTGATGTTCGGACTGAACCGAATCCGATGTCATCACG
AGACCCAAGAATAGCGCCACATGATCCGTTGAAGTTAGGGTGTGAGAAACATGGTATGCCATGTTCTCCATTTAATCGAA
AACATTTGGAATTAGCAACAACTCATTTAAAGGAGAAGTTAATTTCCGTAGTTAAACCTATAAACGGATGCAAGATTAGA

AGTTTGCAAGATGCTGTGTGTGGTGTACCAGGTTTGGATGGCTTTGATTCAATATCCTGGAATACTAGTGCTGGTTTTCC
TTTATCTTCATTAAAACCACCAGGCTCTTCTGGTAAGCGATGGTTGTTTGATATTGAATTACAAGATTCAGGATGTTATC
TTTTGAGAGGGATGAGACCTGAACTTGAGATACAGTTGACAACAACTCAGTTAATGAGGAAGAAGGGAATAAAGCCTCAC
ACTATATTCACGGATTGTTTAAAAGATACATGTTTGCCTGTGGAAAAATGCAGAATACCTGGTAAGACTAGAATATTTAG
TATAAGTCCCGTCCAATTTACGATTCCATTCCGACAATACTATCTCGATTTTATGGCGTCGTACCGTGCCGCTAGACTTA
ATGCTGAGCATGGAATAGGTATAGACGTGAACAGCTTGGAGTGGACAAACTTGGCAACAAGTCTGTCGAAGTATGGCACG
CATATTGTGACAGGAGATTACAAGAATTTTGGTCCTGGGTTAGATTCTGATGTTGCCGCTTCAGCTTTCGAAATTATCAT
TGATTGGGTGTTAAATTACACTGAAGAAGATGATAAAGACGAAATGAAGCGTGTAATGTGGACTATGGCTCAGGAAATTT
TAGCTCCTAGTCACTTATGTCGTGATTTAGTATATCGCGTACCATGCGGTATTCCTTCTGGATCACCAATTACGGACATT
TTGAATACTATTTCGAATTGTTTGTTAATTCGATTGGCTTGGCAAGGTATTACCGATTTGCCTTTATCCGAATTTTCTAG
ACATGTCGTGCTAGTTTGTTACGGTGATGATCTCATCATGAATGTAAGTGATGAAATGATAGACAAATTCAACGCTGTAA
CAATTGGTGATTTCTTTTCGCGATATAAGATGGAATTTACGGATCAGGATAAATCTGGAAATACAGTGCGGTGGCGAACT
TTACAAACTGCCACCTTTTTGAAGCATGGGTTCTTGAAACATCCAACAAGACCCGTGTTTCTAGCCAATCTGGATAAGGT
TTCTATAGAAGGAACAACCAATTGGACACATGCTCGAGGATTGGGTCGTCGAGTAGCAACCATTGAGAATGCTAAACAAG
CGCTAGAGTTGGCATTCGGATGGGGTCCCGAATACTTTAATCATGTTCGGAATACCATTAAAATGGCATTCGACAAGTTA
GGTATTTATGAGGACCTCATCACATGGGAAGAAATGGATGTTAGATGTTATGCTAGCGCGTAATTTTAAGATTTTAATAC
TCATTAAAATTAATTTATATTTAGGTTATTGGAATTGAGGGAAGTACCACCCCCCAAGACCTTCGTTTTAAATCTACTAA
GAGGAGTGAACTTGCATATAAGAGTCTAAAAGCAGAGTGGATTAGACCACCACTTTTAGCTTATATGTGAGGAAGGTTGA
GTTGCCTCTAAAGACTCAGCTCCGTAGTAGAGTAGTTTTAGTTACGATTAAAGTGGTACTCTAGGTTAGGTGTTACTCGC
GTATTGTCGCATAACGGCAATGCGTCCTAATTTTAGTATAGTTTAACCATAATAGTAAAAAAAAAAAAAAAAAAAAA

> Code of pupa: D4-DWV-A; IVA partial genome DWV-B contig sequence
(Row 11 of Figure S5, code: DWV-B_contig.00004-D4-A)

TTGATTTTGAAGGTAAATATATATAAAAATGGCATTTAGTTGTGGAACTCTTTCTTATGCTGCTGTTGCCCAAGCTCCCT
CTGTAGCTCATGCTCCCCGTAGTTGGGAGATTGATGAAGCTAGGCGTCGACGCGTTATTAAGCGTTTGGCGTTGGAACAG
GAACGGATTCGAAACGTTCTTGACGTCACTGTGTATGATCATACAACGTGGGAGCAAGAAGATGCGCGTGATAATGAGTT
CCTTACGGAACAATTGAATAATTTATATACGATATATTCTATAGCTGAAAGATGTACCCGCCGTCCTGTTCAAGAACATG
TCCCCATTTCAATCAGTAATAGATATTCCCCTTTAGAATCCCTTAAGATTGAGGTAGGAAAAGACGCGGGTGAGTTCGTA
TTTAAGAAACCCAAATATACAAAGATTTGTAAGAAAGTGAAACGGGTGGCATCAAAATTTGTGCGCGAGAAAGTTGTTAG
GCCCGTTTGTAATCGATCGCCTATGTTATTATTTAAAATTAAGAAAGTAATATATGATTTACATTTGTATCGGTTACGGA
AACAAGTTCGGCTTCTCAGACGCGAAAAACAGCGTGAATACGAGTTAGAGTGTGTTACTAGTTTGCTACAGCTATCTAAT
CCTGTTTCAGCTAAACCTGAGATGGACAATCCTAATCCTGGTCCAGATGGTGAAGGTGAAGTTGAATTAGAAAAGGATAG
TAATGTAGTATTAACTACACAACGTGATCCTAGTACCTCTATTCCTGCTCCAACTAGTGTGAAGTGGAGTAGATGGACTA
GTAATGATGTTGTGGATGATTATGCCACTATAACTTCGCGCTGGTATCAAATTGCCGAATTTGTATGGTCAAAGGATGAT
CCATTTGATAAGGAATTGGCGCGCTTAATTTTACCTCGAGCTTTGTTATCTAGTATTGAGGCTAATTCTGACGCTATTTG
TGATGTACCTAATACTATTCCGTTTAAGGTACATGCATATTGGCGTGGAGATATGGAAGTTCGAGTGCAGATTAACTCGA
ATAAATTCCAGGTTGGTCAATTACAGGCAACTTGGTACTATTCGGATCATGAAAATTTGAATATTCAGACGAAGCGAAGT
GTGTATGGTTTTTCGCATATGGATCATGCTCTGATTAGCGCATCAGCGAGTAATGAAGCAAGATTAGTGATACCTTTTAA
ACACGTATATCCATTCTTACCAACGCGTGTCGTTCCTGATTGGACAACTGGTATTCTTGATATGGGTACCTTAAATATTC
GTGTAATTGCTCCACTACGTATGAGTGCGACGGGACCAACCACTTGTAATGTTGTAGTATTTATTAAGTTAAATAATAGT
GAATTCACTGGTACTTCTTCTGGTAAGTTTTACGCAAATCAAATTAGGGCAAAACCTGAAATGGACCGTGTGTTAAATTT
GGCAGAAGGATTACTAAATAATACTGTAGGTGGTTGTAATATGGATAATCCGTCATATCAGCAATCTCCGCGTCATTTTG
TTCCTACTGGTATGCATAGTTTAGCTTTAGGCACTAATTTAGTAGAGCCTTTGCATGCATTACGATTAGATGCATCAGGT
ACAACACAACATCCAGTTGGGTGTGCGCCTGATGAAGATATGACTGTATCTTCCATTGCATCACGATATGGTTTAATTCG
CCAAGTGCAATGGAAGAAAGACCATGCGAAAGGATCATTATTATTACAACTTGATGCTGATCCTTT

> Code of pupa: D4-DWV-A; IVA partial genome DWV-B contig sequence
(Row 12 of Figure S5, code: DWV-B_contig.00005-D4-A)

GGGTAGGTTAATTGTAGGTTATGTTCCTGGACTGACTGCTTCTTTACAACGTCAAATGGACTATATGAAATTGAAGTCAT
CTAGTTATGTGGTGTTTGATTTACAGGAAAGTAATAGTTTTACGTTTGAAGTGCCCTATGTGTCATACAGACCGTGGTGG
GTGCGTAAGTATGGTGGTAATTATCTACCATCCTCTACTGATGCGCCTAGCACACTGTTTATGTATGTACAAGTACCATT
GATACCTATGGAAGCTGTTTCTGATACTATAGATATCAATGTGTATGTGCGTGGTGGCAGTTCGTTTGAGGTTTGTGTTC
CAGTCCAACCTAGTTTAGGTTTGAACTGGAATACAGATTTCATATTACGTAATGATGAGGAGTACCGCGCAAAGAATGGA
TATGCACCATATTATGCTGGTGTGTGGCATAGCTTCAATAATAGTAATTCGCTTGTTTTTAGATGGGGTTCGGCTTCAGA
TCAAATTGCTCAATGGCCAACAATAACAGTGCCTCGAGGAGAGTTGGCATTCCTGCGTATCCGCGATGCTAAGCAAGCTG
CTGTAGGAACGCAACCTTGGCGTACTATGGTCGTTTGGCCTTCAGGTCATGGATATAACATTGGAATACCAACTTATAAT
GCTGAACGAGCAAGACAACTTGCTCAGCATTTGTATGGTGGTGGGTCTTTGACAGATGAAAAGGCTAAGCAATTATTTGT
GCCTGCTAACCAGCAAGGACCCGGCAAAGTAAGTAATGGTAACCCCGTCTGGGAAGTAATGCGCGCGCCTCTTGCAACTC
AGCAAGCGCATATACAAGATTTTGAATTTGTTGAAGCTGTTCCAGAAGGCGAAGAATCACGCAACACTACGGTGCTAGAC
ACGACAACAACGTTACAGTCTAGCGGATTTGGTCGCGCTTTCTTCGGTGAGGCATTTAACGATCTTAAGACGTTAATGCG
CCGATACCAATTATATGGTCAATTATTGTTATCCGTTACTACGGATAAGGATATTGATCATTGTATGTTTACCTTCCCTT

GTTTACCTCAAGGGTTAGCGTTAGATATAGGTTCGGCTGGATCTCCTCATGAAATATTTAATCGCTGCCGTGATGGTATC
ATTCCATTGATAGCGTCAGGGTATCGGTTTTATCGAGGCGATTTACGGTTCAAAATTGTTTTCCCAAGTAACGTTAATAG
CAATATTTGGGTACAACATCGACCAGATCGTAGACTGAAAGGATGGTCTGAAGCGAAAATAGTAAACTGTGATGCTGTAT
CTACTGGACAAGGTGTTTATAATCATGGATATGCTAGTCATATTCAGATTACGCGTGTAAATAATGTTATAGAATTGGAA
GTTCCGTTTTATAACGCTACGTGCTATAATTATTTGCAAGCGTTTAACCCATCTAGTGCAGCGTCGAGTTATGCCGTTTC
GCTCGGAGAGATTTCGGTTGGTTTTCAAGCTACTAGTGAT

> Code of pupa: D4-DWV-A; IVA partial genome DWV-B contig sequence (Row 13 of Figure S5, code: DWV-B_contig.00002-D4-A)

CCATCTAGTGCAGCGTCGAGTTATGCCGTTTCGCTCGGAGAGATTTCGGTTGGTTTTCAAGCTACTAGTGATGACATTGC
AGCCATAGTTAATAAACCTGTAACTATATATTACAGTATTGGCGATGGTATGCAGTTTTCGCAGTGGGTTGGTTATCAAC
CAATGATGATTCTAGATCAATTGCCAGCACCAGTAGTTAGGGCTGTGCCTGAGGGCCCTATAGCTAAGATAAAGAACTTT
TTCCACCAAACGGCAGATGAAGTTCGAGAAGCTCAGGCCGCAAAGATGCGTGAAGATATGGGTATAGTAGTCCAAGACGT
CATAGGAGAGTTAAGTCAGGCTATACCCGATCTTCAACAACCGGAAGTTCAAGCGAATGTTTTTTCTCTGGTGTCACAGT
TAGTGCATGCTATCATCGGTACTAGTCTTAAGACAGTTGCTTGGGCGATTGTTTCGATTTTTGTAACTTTAGGTTTGATT
GGACGTGAAATGATGCATTCAGTCATAACTGTAGTTAAGCGGTTATTAGAAAAATATCACTTGGCGACGCAACCCCAGGA
ATCCGCCAATTCAGGTACGGTTATTTCCGCTATTCCAGAAGCACCCAATGCTGAAGCAGAGGAGGCCAGTGCCTGGGTAT
CCATTATTTATAATGGTGTGTGTAATATGTTGAATGTAGCCGCTCAAAAACCGAAACAATTTAAAGATTGGGTAAAATTA
GCTACCGTAGATTTTAGTAATAATTGTAGAGGTAGTAATCAGGTATTTGTGTTTTTCAAGAATACGTTTGAAGTGTTGAA
GAAGATGTGGGGTTATGTGTTTTGTCAGAGTAATCCTGCAGCGCGACTCTTGAAAGCAGTGAATGATGAACCTGAGATTT
TAAAAGCGTGGGTTAAAGAATGTCTGTATTTAGATGATCCTAAATTTAGAATGCGACGTGCGCATGATCAAGAGTATATT
GAGAGAGTGTTTGCGGCCCATTCGTATGGACAAATTTTATTGCATGACTTAACGGCTGAAATGAATCAATCGCGTAATTT
AAGTGTGTTTACGAGAGTGTACGATCAAATATCTAAATTGAAGACGGATCTCATGGAAATGGGATCAAACCCATATATCA
GGCGTGAATGCTTTACGATTTGTATGTGTGGTGCATCTGGAATTGGTAAGTCTTATTTAACTGATTCTTTATGCAGCGAG
CTCTTACGTGCGAGTCGTACTCCAGTGACAACGGGCATTAAGTGTGTCGTGAACCCTTTGTCTGATTATTGGGATCAGTG
TGATTTTCAGCCCGTTTTATGTGTTGATGACATGTGGAGTGTTGAAACGTCTACTACGCTCGATAAACAGTTAAATATGC
TATTTCAGGTTCATTCACCAATTGTACTTTCACCTCCTAAAGCTGATTTAGAAGGTAAGAAAATGCGTTATAATCCTGAA
ATATTCATATATAATACGAATAAACCTTTTCCGAGGTTTGATCGTATAGCTATGGAAGCTATTTATCGACGTAGAAACGT
TTTAATTGAATGTAAGGCTAATGAAGAGAGGAAGCGTGGATGTAAACATTGTGAGAATAATATACCCATTGCTGAATGTA
GTCCAAAAATTTTGAAAGATTTTCATCACATTAAATTTCGTTATGCTCATGATGTGTGTAATTCTGAAACTACGTGGTCT
GAGTGGATGTCGTATAATGAATTTTTGGAATGGATTACTCCCGTATATATGGCTAATCGACGTAAAGCAAATGAATCGTT
TAAGATGCGTGTTGATGAAATGCAAATGTTGCGTATGGATGAGCCCTTGGAAGGCGATAATATTTTAAATAAGTATGTTG
AAGTTAATCAGCGCTTAGTTGAGGAAATGAAAGCTTTTAAAGAGCGAACCCTCTGGGCTGATTTACAACGTGTTGGCTCA
GAGATTAGTACTTCAGTTAAGAAAGCATTACCAACTATTTCCATTACTGAGAAGCTACCACATTGGACTATCCAATGTGG
CATAGCTAAGCCTGAAATGGATCATGCTTATGAAGTTATGAGTTCATATGCAGCAGGAATGAACGCAGAAATTGAAGCGC
ATGAACAAGTTCGTCGTTCTTCTTTGGAATGTCAGTGTATTGAGCCCTCAACTTCAAGACCTCTGGATGAAGAGGGTCCT
ACTATCGACGAGGAATTACTTGGCGAAGTAGAATTTACTTCTTCAGCTTTGGAGCGTTTGGTTGATGAAGGGTATATTAC
TGGTAAACAAAAGAAGTACATGGCAACTTGGTGTACGAAACGAAGAGAGCATGTATCCGATTTTGATTTAGTATGGACGG
ATAATTTGCGTGTTTTGAGTGCGTATGTCCACGAGCGTTCTACATCTACGCGTTTATCTACCGATGATGTTAAATTATTT
AAGACGATTAGTATGTTACATCAGAGGTATGACACCACTGATTGTGCAAAATGCCAACATTGGTATGCACCATTAACAGC
TATTTATGTTGATGATAGAAAGCTATTTTGGTGCCAGAAGGAGACTAAGACTTTGATAGATGTTCGTAAATTGTCGAAAG
AGGACGTTACAGTCCAATCGAAATTAATTAACTTATCGGTTCCGTGCGGTGATGTATGTATGTTACATTCTAAGTACTTT
AATTATTTATTCCATAAAGCGTGGTTGTTTGAAAATCCAACATGGCGTTTAATATATAATGGTACTAAGAAAGGTATGCC
TGAGTATTTCATGAATTGCGTGGATGAAATTTCATTAGATTCAAAATTTTGTAAAGTAAAGGTTTGGCTTCAAGCAATTA
TTGATAAATATTTGACTCGTCCAGTGAAAATGATTCGTGACTTTCTATTTAAATGGTGGCCGCAAGTAGCATACGTGTTA
AGTTTGTTAGGTATAATTGGTATAACTGCGTATGAGATGCGTAATCCTAAATCAACAGCAGAAGACTTGGCTGAGCACTA
TGTTAATAGGCATTGTAGTTCAGATTTTTGGTCACCAGGTATGGCGACTCCTCAGGGATTAAAATATAGTGAAGCGATAA
CAGCTAAAGCGCCTAGAATCCATAGATTGCCCGTTACTACTAGACCTCAGGGATCAACGCAACAAGTTGACGCCGCTGTG
AATAAGATTTTGCAGAATATGGTGTATATCGGTGTTGTGTTTCCGAAAGTGCCTGGTAGTAAGTGGCGAGATATTAATTT
TAGATGTCTTATGCTTCATAATCGGCAATGTTTGATGTTGCGGCATTACATTGAGTCGACGGCTGCTTTTCCGGAGGGTA
CCAAATACTATTTTAAGTATATCCATAATCAAGAAACTCGAATGTCAGGTGATATATCTGGTATTGAGATTGATTTATTG
AGTTTGCCTAGATTGTATTATGGTGGCTTAGCTGGGGAAGAGTCGTTCGATAGCAATATAGTGTTAGTAACTATGCCGAA
TAGAATTCCTGAGTGTAAGAGTATTGTGAAGTTTATAGCTTCACATGCTGAACATGCTCGTGCTCAAAATGATGGTGTGT
TAGTTACTGGTGAACATACTCAGTTATTGGCGTTCGAGAATAATAATAAAACACCTATAAGTATTAATGCTGATGGT

> Code of pupa: D4-DWV-A; IVA partial genome DWV-B contig sequence (Row 14 of Figure S5, code: DWV-B_contig.00003-D4-A)

CGTGCTCAAAATGATGGTGTGTTAGTTACTGGTGAACATACTCAGTTATTGGCGTTCGAGAATAATAATAAAACACCTAT
AAGTATTAATGCTGATGGTTTGTATGAGGTTATACTTCAAGGAGTATACACTTATCCATACCATGGTGATGGTGTTTGTG
GGTCTATATTATTGTCTCGTAATTTACAACGCCCGATTATAGGGATCCATGTAGCTGGTACTGAAGGATTACATGGCTTT
GGTGTTGCTGAACCTCTTGTTCATGAGATGTTCACTGGGAAAGCAATAGAGAGTGAAAGGGAACCGTATGATCGTGTGTA

TGAATTACCTTTGCGTGAATTAGATGAATCTGATATAGGTTTAGATACTGATTTATATCCTATAGGAAGAGTTGATGCGA
AATTAGCTCATGCCCAAAGTCCTTCAACAGGAATTAAAAAGACGCTTATTCATGGTACTTTTGATGTTCGGACTGAACCG
AATCCGATGTCATCACGAGACCCAAGAATAGCGCCACATGATCCGTTGAAGTTAGGGTGTGAGAAACATGGTATGCCATG
TTCTCCATTTAATCGAAAACATTTGGAATTAGCAACAACTCATTTAAAGGAGAAGTTAATTTCCGTAGTTAAACCTATAA
ACGGATGTAAGATTAGAAGCTTGCAAGATGCTGTGTGTGGTGTACCAGGTTTGGATGGTTTTGATTCAATATCCTGGAAT
ACTAGTGCTGGTTTTCCTTTATCTTCATTAAAACCGCCAGGCTCTTCTGGTAAGCGATGGTTGTTTGATATTGAATTACA
AGATTCAGGATGTTATCTTTTGAGAGGGATGAGACCTGAACTTGAGATACAGTTGACAACAACTCAGTTAATGAGGAAGA
AGGGAATAAAGCCTCACACTATATTCACGGATTGTTTGAAAGATACATGTTTGCCTGTGGAAAAATGTAGAATACCTGGT
AAGACTAGAATATTTAGTATAAGTCCCGTCCAATTTACGATTCCATTCCGACAATACTATCTCGATTTTATGGCGTCGTA
CCGTGCCGCTAGACTTAATGCTGAGCATGGAATAGGTATAGACGTGAACAGCTTGGAATGGACAAACTTGGCAACAAGTC
TGTCGAAGTATGGCACGCATATTGTGACAGGAGATTATAAGAATTTTGGTCCTGGGTTAGATTCTGATGTTGCCGCTTCA
GCTTTCGAAATTATCATTGATTGGGTGTTAAATTACACTGAAGAAGATGATAAAGACGAAATGAAGCGTGTAATGTGGAC
TATGGCTCAGGAAATTTTAGCTCCTAGTCACTTATGTCGTGATTTAGTATATCGCGTACCATGCGGTATTCCTTCTGGAT
CACCAATTACGGACATTTTGAATACTATTTCGAATTGTTTGTTAATTCGATTGGCTTGGCAAGGTATTACCGATTTGCCT
TTATCCGAATTTTCTAGACATGTCGTGCTAGTTTGTTACGGTGATGATCTCATCATGAATGTAAGTGATGAAATGATAGA
CAAATTCAACGCTGTAACAATTGGTGATTTCTTTTCGCGATATAAGATGGAATTTACGGATCAGGATAAATCTGGAAATA
CAGTGCGGTGGCGAACTTTACAAACTGCCACCTTTTTGAAGCATGGGTTCTTGAAACATCCAACAAGACCCGTGTTTCTA
GCCAATCTGGATAAGGTTTCTATAGAAGGAACAACCAATTGGACACATGCTCGAGGATTGGGTCGTCGAGTAGCAACCAT
TGAGAATGCTAAACAAGCGCTAGAGTTGGCATTCGGATGGGGTCCCGAATACTTTAATCATGTTCGGAATACCATTAAAA
TGGCATTCGACAAGTTAGGTATTTATGAGGATCTCATCACATGGGAAGAAATGGATGTTAGATGTTATGCTAGCGCGTAA
TTTTAAGATTTTAATACTCATTAAAATTAATTTATATTTAGGTTATTGGAATTGAGGGAAGTACCACCCCCCAAGACCTT
CGTTTTAAATCTACTAAGAGGAGTGAACTTGCATATAAGAGTCTAAAAGCAGAGTGGATTAGACCACCACTTTTAGCTTA
TATGTGAGGAAGGTTGAGTTGCCTCTAAAGACTCAGCTCCGTAGTAGAGTAGTTTTAGTTACGATTAAAGTGGTACTCTA
GGTTAGGTGTTACTCGCGTATTGTCGCATAACGGCAATGCGTCCTAATTTTAGTATAGTTTAACCATAATAGTAA

Figure S5. Nucleotide alignment of all DWV contigs generated by IVA *de novo* assemblies of all DWV reads in our four NGS datasets. DWV-A Accession No. NC_004830.2 and DWV-B Accession No. NC_006494.1 were used as reference sequences. The McM-2016 sequences correspond to the references sequences of DWV-A and DWV-B generated by McMahon *et al.* (2016), and from which our inocula were derived. Inoculum refers to our inocula (DWV-A or DWV-B), D4-A to a pupa inoculated with inoculum A, and V4-B to a pupa inoculated with inoculum B. The grey scale colours indicate percent identity between all simultaneously aligned sequences within either DWV-A (rows 1-6) or DWV-B (rows 7-14) (black=100% identity).

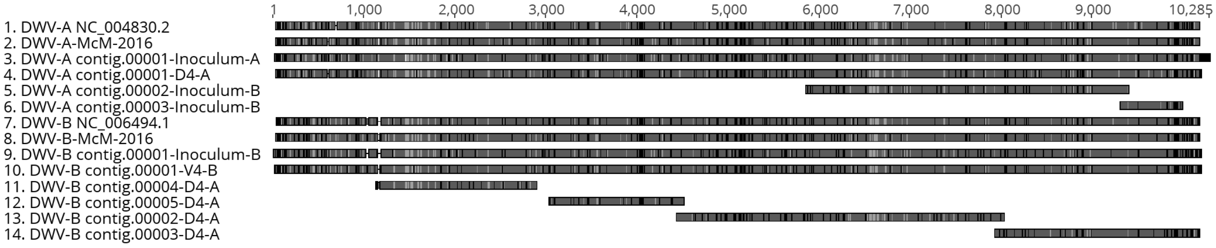

McMahon, D.P.; Natsopoulou, M.E.; Doublet, V.; Fürst, M.; Weging, S.; Brown, M.J.F.; Gogol-Döring, A.; Paxton, R.J. Elevated virulence of an emerging viral genotype as a driver of honeybee loss. *Proceedings of the Royal Society of London B: Biological Sciences* **2016**, *283*, 20160811, doi: 10.1098/rspb.2016.0811

Figure S6. Maximum Likelihood phylogenetic tree of all DWV contig sequences generated by IVA *de novo* assemblies of all DWV reads in our four NGS libraries (taxa 'contigs'). DWV-A Accession No. NC_004830.2 and DWV-B Accession No. NC_006494.1 were used as reference sequences. The McM-2016 sequences correspond to the references sequences of DWV-A and DWV-B generated by McMahon *et al.* (2016), and from which our inocula were derived. Inoculum refers to our inocula (DWV-A or DWV-B), D4-A to a pupa inoculated with inoculum A, and V4-B to a pupa inoculated with inoculum B. The scale bar represents substitution rate per site. Bootstrap values were calculated after 100 replicates using the HKY85 model.

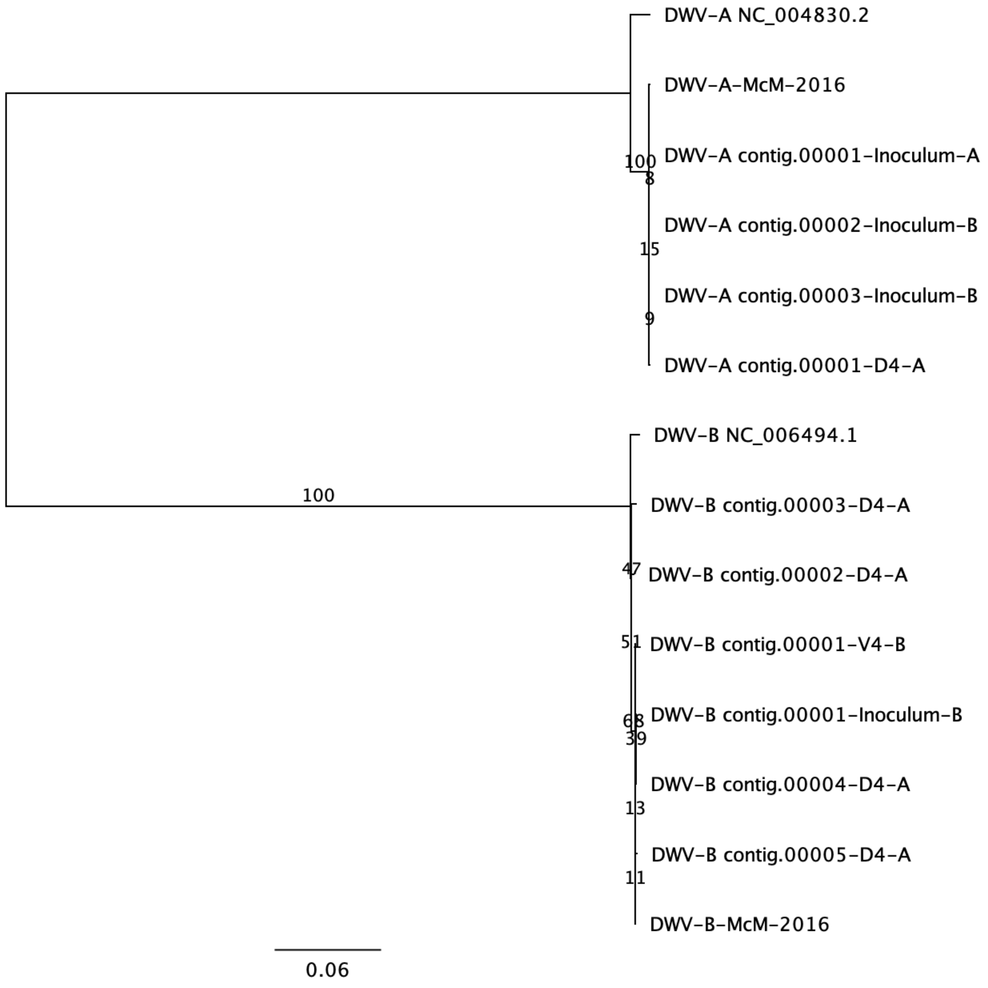

McMahon, D.P.; Natsopoulou, M.E.; Doublet, V.; Fürst, M.; Weging, S.; Brown, M.J.F.; Gogol-Döring, A.; Paxton, R.J. Elevated virulence of an emerging viral genotype as a driver of honeybee loss. *Proceedings of the Royal Society of London B: Biological Sciences* **2016**, *283*, 20160811, doi: 10.1098/rspb.2016.0811

Figure S7. Titres of DWV-A and DWV-B per honey bee pupa at days 0 and 3 post inoculation (p.i.) with either DWV-A or DWV-B (n = 4 per treatment and age class). Box plots show the median (dark bar), upper and lower interquartiles (coloured box), and 95% confidence limits (whiskers). Within a viral genotype, titres did not differ between bees injected with 10^2 or 10^4 virus particles (GLMs $p > 0.05$). The DWV-A titre after 3 days was higher than the DWV-B titre (GLM $p < 0.01$).

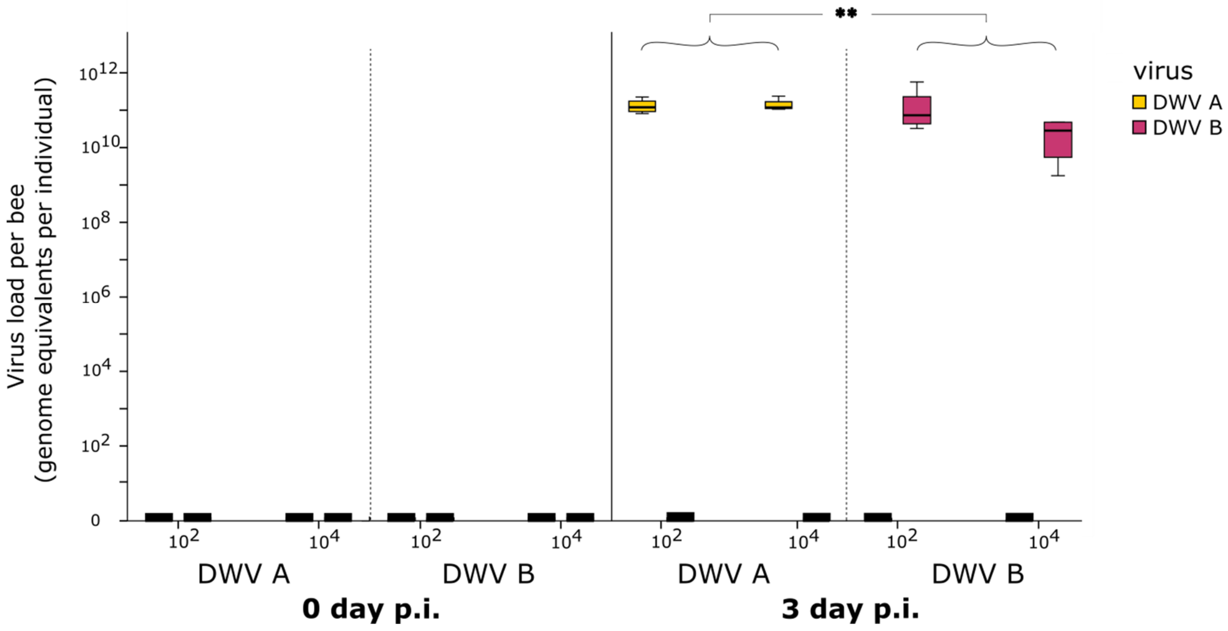

Figure S8. Survival (a) and wing deformities (b) in treatment groups split by colony of origin (colony 1, colony 2). Values above histograms give the number of individuals exhibiting the trait/total number of individuals. C represents the control inoculation devoid of virus.

(a)

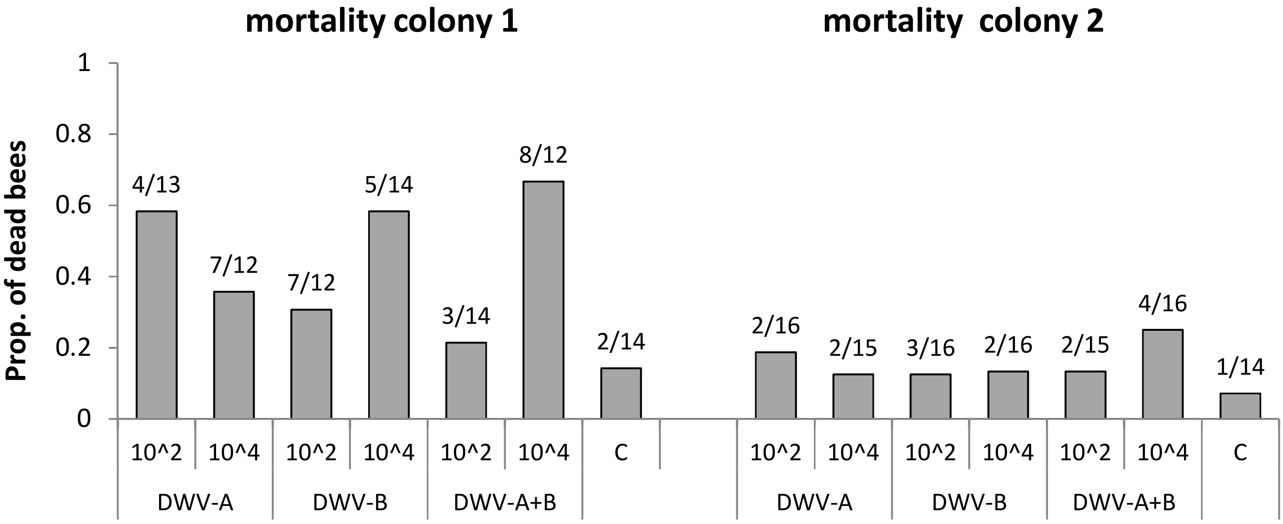

(b)

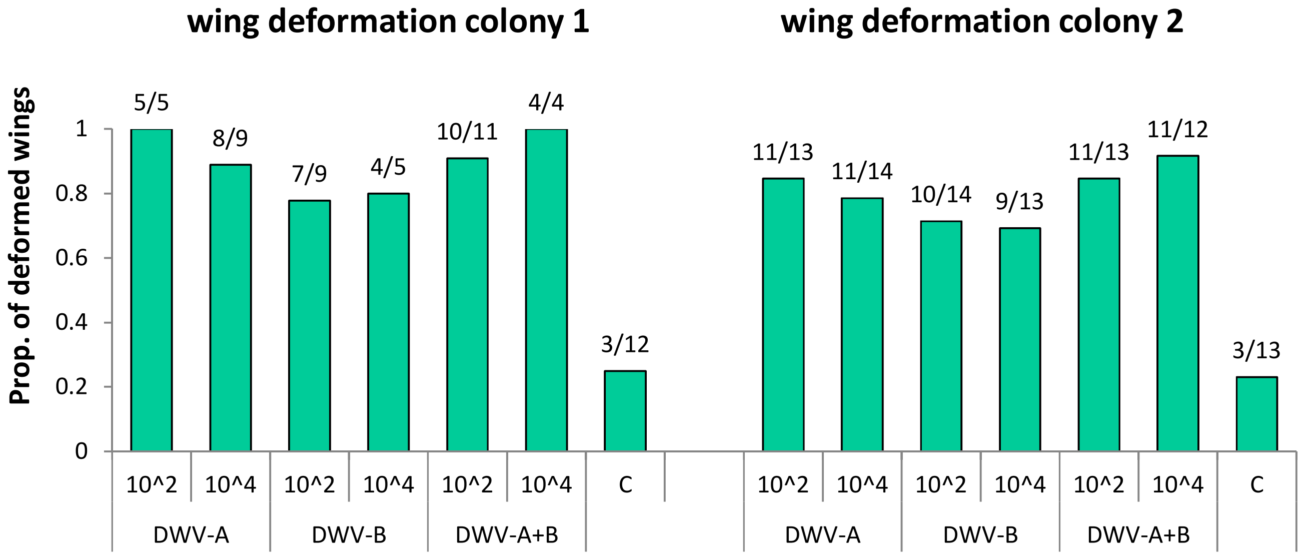

Figure S9. Viral titres per bee in eclosing honey bee adults that as white-eyed pupae had been inoculated with DWV. Data from all our viral treatments (A, B, A+B) were used to generate mean titres of DWV-A and DWV-B, and partitioned by whether the eclosing bee had normal or deformed wings. Only those bees where the experimental infection worked successfully and the bees therefore showed virus titres above 10^7 genome equivalents (Ct <33) were included (n = 4 of 44 bees were excluded, including one bee inoculated with A, one with B and two with A+B). There was no difference in titre between bees with normal *versus* deformed wings for DWV-A (GLMM, z = -0.648, p = 0. 517) or for DWV-B (GLMM, z = -0.764 p = 0.445). Viral titres (DWV-A, DWV-B) separated by viral treatment are given in Figure 4.

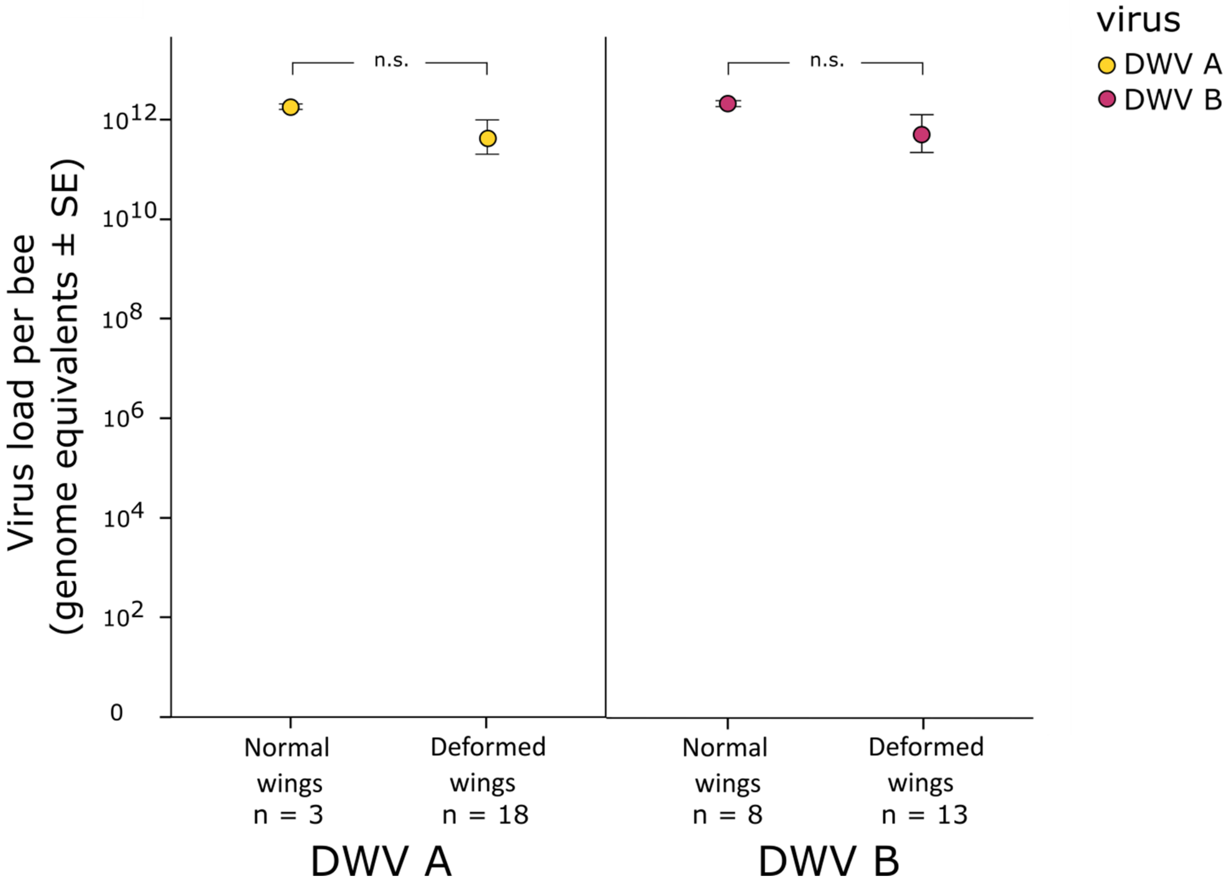

Table S1. Contents (number and % of all NGS reads that could be mapped to contigs or that remained unmapped) of the two NGS libraries generated using either the DWV-A or the DWV-B inoculum.

		**DWV-A inoculum**		**DWV-B inoculum**	
Origin		N reads	**% reads**	N reads	**% reads**
Apis		12,868,152	**86.794**	21,763,325	**67.855**
DWV					
	DWV-A	1,843,693	**12.435**	6,143	**0.019**
	DWV-B	65,169	**0.440**	10,236,879	**31.917**
Other					
	Apis rhadbovirus-1	6,113	**0.041**	33,735	**0.105**
	Other Eukaryota	4,064	**0.027**	6,347	**0.020**
	Bacteria	3,630	**0.024**	6,308	**0.020**
	Other (viral or Eukaryota)*	999	**0.007**	945	**0.003**
	Unknown reads §	35,240	**0.238**	20,628	**0.064**
Total		14,826,061		32,073,365	

*; short contigs that either represent fragments of DNA viruses or eukaryote genes e.g. annotated *Apis* rRNA genes

§; includes NGS reads that could not be assembled into contigs

Table S2. Contents (number and % of all NGS reads that could be mapped to contigs or that remained unmapped) of the two NGS libraries generated from eclosing honey bees that had been inoculated as white-eyed pupae with either the DWV-A (individual pupal code: D4-DWV-A) or the DWV-B inoculum (individual pupal code: V4-DWV-B).

		**D4-DWV-A pupa (DWV-A inoculated)**		**V4-DWV-B pupa (DWV-B inoculated)**	
Origin		N reads	**% reads**	N reads	**% reads**
Apis		11,856,745	**51.955**	7,017,221	**41.870**
DWV					
	DWV-A	9,923,721	**43.484**	969	**0.006**
	DWV-B	30,786	**0.135**	9,538,992	**56.916**
Other					
	Apis rhadbovirus-1	16	**<0.001**	0	
	Other Eukaryota	34,225	**0.150**	22,893	**0.137**
	Bacteria	18,221	**0.080**	15,835	**0.094**
	Other (viral or Eukaryota)*	0		35	**<0.001**
	Unknown §	957,677	**4.196**	163,749	**0.977**
Total		22,821,391		16,759,694	

*; short contigs that either represent fragments of DNA viruses or eukaryote genes e.g. annotated *Apis* rRNA genes

§; includes reads that could not be assembled into contigs

Table S3. Comparison of published consensus sequences for DWV-A (NC_004830.2) and DWV-B (NC_006494.1), inocula A and B of McMahon *et al.* (2016; McM-2016-A and McM-2016-B respectively), those used here (DWV-A inoculum /DWV-B inoculum, which were derived from those of McMahon *et al.* 2016) and virus derived from experimentally inoculated host pupae as they eclosed as adults (individual pupal codes: D4-DWV-A and V4-DWV-B for DWV-A and DWV-B inoculated pupae respectively).

		DWV-A			
		NC_004830.2	McM-2016-A	DWV-A inoculum	D4-DWV-A
DWV-A	NC_004830.2		**195 + 69N**	**194 + 69N**	**195 + 69N**
	McM-2016-A	97.732%		**1**	**2**
	Inoculum A	97.742%	99.990%		**2**
	D4-DWV-A	97.732%	99.980%	99.980%	

		DWV-B			
		NC_006494.1	McM-2016-B	DWV-B inoculum	V4-DWV-B
DWV-B	NC_006494.1		**75 (1 insert)**	**77 (1 insert)**	**75 (1 insert)**
	McM-2016-B	99.258%		**3**	**1**
	Inoculum B	99.239%	99.970%		**5**
	V4-DWV-B	99.258%	99.990%	99.951%	

Upper right diagonal: number of nucleotide base differences
Lower left diagonal: % nucleotide identity
N: bases whose identity could not be unambiguously determined within the sequences

McMahon, D.P.; Natsopoulou, M.E.; Doublet, V.; Fürst, M.; Weging, S.; Brown, M.J.F.; Gogol-Döring, A.; Paxton, R.J. Elevated virulence of an emerging viral genotype as a driver of honeybee loss. *Proceedings of the Royal Society of London B: Biological Sciences* **2016**, *283*, 20160811, doi: 10.1098/rspb.2016.0811
